# Supplementary material for: Influence of biosilica treatments and storage receptacles on the quality of maize (Zea mays L.) and common bean (Phaseolus vulgaris L.) seeds during long-term storage
Source: PLoS One. 2026 Mar 11;21(3):e0344033. doi: 10.1371/journal.pone.0344033 (PMC12978491; doi:10.1371/journal.pone.0344033)
Supplement: S2 Table — (DOCX) [file pone.0344033.s002.docx]

**Influence of biosilica treatments and storage receptacles on the quality of maize (*Zea mays* L.) and common bean (*Phaseolus vulgaris* L.) seeds during long-term storage**

Bertrand Zing Zing ^1,2*^, Charles Rostand Mvongo Mvodo ^1^, Valteri Audrey Voula ^1^, Lin Marcellin Messi Ambassa ^1^, Eugene Ejolle Ehabe ^1^, Placide Desiré Belibi Belibi ^3^, Charles Melea Kede ^2^

^1^ Directorate of Scientific Research, Institute of Agricultural Research for Development, P.O. Box 2123, Yaoundé, Cameroon.

^2^ Laboratory of Chemical and Industrial Bioprocess Engineering, National Higher Polytechnic School of Douala, University of Douala, P.O. Box 2701, Douala, Cameroon.

^3^ Department of Inorganic Chemistry, University of Yaoundé I, P.O. Box 812, Yaoundé, Cameroon.

∗ Corresponding author e-mail address: [zingbertrand29@gmail.com](mailto:zingbertrand29@gmail.com) (B.Z.Z)

Bertrand Zing Zing: <https://orcid.org/0000-0002-3892-8950>.

Eugene Ejolle Ehabe: <https://orcid.org/0000-0003-2215-2112>.

Charles Melea Kede: <https://orcid.org/0000-0002-4951-3152>.

**Table 1**. Post-hoc analysis layout for maize and common bean cultivars

| 1. Maize species (CMS 8501 and CMS 8704) | | | | | | | | | | | | | | |
| --- | --- | --- | --- | --- | --- | --- | --- | --- | --- | --- | --- | --- | --- | --- |
| *Source of variations* | *Somme of Square* | | | *DL* | | | *Mean of square* | | *F-Value* | | *P>F* | | *F-crit.* | |
| Between groups | 869426.982 | | | 5 | | | 173885.396 | | 321.844581 | | 3.3497E-7 | | 4.38737419 | |
| Within groups | 3241.6652 | | | 6 | | | 540.277533 | |  | |  | |  | |
|  |  | | |  | | |  | |  | |  | |  | |
| Total | 872668.647 | | | 11 | | |  | |  | |  | |  | |
| 1. Common bean species (FEB 190 and NUV6) | | | | | | | | | | | | | |  |
| *Source of variations* | | *Somme of Square* | *DL* | | | *Mean of square* | | *F-Value* | | *P>F* | | *F-crit.* | |  |
| Between groups | | 1144176.72 | 5 | | 228835.345 | | | 188.763458 | | 1.6425E-06 | | 4.38737419 | |  |
| Within groups | | 7273.7175 | 4 | | 1212.28625 | | |  | |  | |  | |  |
|  | |  |  | |  | | |  | |  | |  | |  |
| Total | | 1151450.44 | 11 | |  | | |  | |  | |  | |  |
