# Supplementary material for: Influence of biosilica treatments and storage receptacles on the quality of maize (Zea mays L.) and common bean (Phaseolus vulgaris L.) seeds during long-term storage
Source: PLoS One. 2026 Mar 11;21(3):e0344033. doi: 10.1371/journal.pone.0344033 (PMC12978491; doi:10.1371/journal.pone.0344033)
Supplement: S4 Table — (DOCX) [file pone.0344033.s004.docx]

**Influence of biosilica treatments and storage receptacles on the quality of maize (*Zea mays* L.) and common bean (*Phaseolus vulgaris* L.) seeds during long-term storage**

Bertrand Zing Zing ^1,2*^, Charles Rostand Mvongo Mvodo ^1^, Valteri Audrey Voula ^1^, Lin Marcellin Messi Ambassa ^1^, Eugene Ejolle Ehabe ^1^, Placide Desiré Belibi Belibi ^3^, Charles Melea Kede ^2^

^1^ Directorate of Scientific Research, Institute of Agricultural Research for Development, P.O. Box 2123, Yaoundé, Cameroon.

^2^ Laboratory of Chemical and Industrial Bioprocess Engineering, National Higher Polytechnic School of Douala, University of Douala, P.O. Box 2701, Douala, Cameroon.

^3^ Department of Inorganic Chemistry, University of Yaoundé I, P.O. Box 812, Yaoundé, Cameroon.

∗ Corresponding author e-mail address: [zingbertrand29@gmail.com](mailto:zingbertrand29@gmail.com) (B.Z.Z)

Bertrand Zing Zing: <https://orcid.org/0000-0002-3892-8950>.

Eugene Ejolle Ehabe: <https://orcid.org/0000-0003-2215-2112>.

Charles Melea Kede: <https://orcid.org/0000-0002-4951-3152>.

**Table 3.** Means of common beans and maize damaged after treatment with biosilica and stored for 06 months

| **Cultivars** | **Months of storage** | **Percent damaged grains (%)** | | | | | | |  |  |  |
| --- | --- | --- | --- | --- | --- | --- | --- | --- | --- | --- | --- |
|  |  | **Glass jars (GJ)** | | **Check (GJ)** | | **Polypropylene (PP)** | **Check (PP)** | **Polyethylene (PE)** | **Check (PE)** | |  |
| **CMS 8501** | **August** | | 1,2 ± 0,5^c^ | 2,98 ± 0,01^e^ | | 0,515 ± 0,001^d^ | 0,512 ± 0,001^f^ | 0,41 ± 0,04^c^ | 2,09 ± 0,06^c^ | |  |
|  | **September** | | 12 ± 2^c^ | 13,08 ± 0,02^d^ | | 5,5 ± 0,6^d^ | 5,320 ± 0,004^e^ | 1,60 ± 0,06^b,c^ | 2,75 ± 0,08^d^ | |  |
|  | **October** | | 37 ± 12^b^ | 40,41 ± 0,05^c^ | | 21 ± 2^c^ | 13,6 ± 0,1^d^ | 2,19 ± 0,06^d^ | 11,4 ± 0,7^b^ | |  |
|  | **November** | | 41 ± 8^b^ | 43,22 ± 0,06^b^ | | 22 ± 4^c^ | 23,43 ± 0,02^c^ | 4,58 ± 0,02^a^ | 17,57 ± 0,05^b^ | |  |
|  | **December** | | 41 ± 8^b^ | 43,4 ± 0,4^b^ | | 32 ± 7^b^ | 38,34 ± 0,01^b^ | 2,1 ± 0,6^b^ | 17,7 ± 0,3^b^ | |  |
|  | **January 2024** | | 100 ± 0^a^ | 100 ± 0^a^ | | 89,45 ± 0,03^a^ | 90,47 ± 0,01^a^ | 5 ± 1^a^ | 100,00 ± 0,05^a^ | |  |
|  | **F-values** | | 78,27 | 2300,40 | | 265,81 | 77709,13 | 1183,14 | 51950,82 | |  |
|  | **P˃F** | | ˂0,0001 | ˂0,0001 | | ˂0,0001 | ˂0,0001 | ˂0,0001 | ˂0,0001 | |  |
| **CMS 8704** | **August** | | 0,8 ± 0,4^e^ | 1,245 ± 0,005^e^ | | 0,437 ± 0,40^e^ | 0,437 ± 0,001^e^ | 2,08 ± 0,02^c^ | 3,1 ± 0,2^e^ | |  |
|  | **September** | | 15 ± 3^d^ | 21,776 ± 0,003^d^ | | 11,8 ± 0,4^d,e^ | 16,6 ± 0,2^d^ | 16,8 ± 0,7^b^ | 13,81 ± 0,05^d^ | |  |
|  | **October** | | 17,5 ± 0,5^c,d^ | 22,017 ± 0,008^d^ | | 23 ± 3^c,d^ | 33,72 ± 0,01^c^ | 15,36 ± 0,04^b^ | 16,8 ± 0,7^c^ | |  |
|  | **November** | | 24 ± 8^c^ | 31,93 ± 0,05^c^ | | 28,81 ± 0,09^c^ | 37 ± 6^c^ | 17 ± 2^b^ | 24,71 ± 0,02^b^ | |  |
|  | **December** | | 35,8 ± 0,3^b^ | 44 ± 4^b^ | | 49,797 ± 0,005^b^ | 49 ± 3^b^ | 17 ± 3^b^ | 24,72 ± 0,04^b^ | |  |
|  | **January 2024** | | 100,0 ± 0,0^a^ | 100,0 ± 0,0^a^ | | 93 ± 7^a^ | 100,0 ± 0,0^a^ | 100,0 ± 0,0^a^ | 100,00 ± 0,09^a^ | |  |
|  | **F-values** | | 385,50 | 2300,96 | | 148,73 | 562,78 | 31,00 | 50510,81 | |  |
|  | **P˃F** | | ˂0,0001 | ˂0,0001 | | ˂0,0001 | ˂0,0001 | ˂0,0001 | ˂0,0001 | |  |
|  | **August** | | 0 ± 0^b^ | 0 ± 0^d^ | | 0,193 ± 0,001^e^ | 0,483 ± 0,001^f^ | 0,00 ± 0,00^c^ | 0,47 ± 0,02^f^ | |  |
| **FEB-190** | **September** | | 1,3 ± 0,8^b^ | 1,169 ± 0,004^c^ | | 0,91 ± 0,07^d^ | 1,25 ± 0,07^e^ | 0,52 ± 0,07^c^ | 1,1 ± 0,1^d^ | |  |
|  | **October** | | 2,4 ± 0,9^b^ | 2,308 ± 0,002^c^ | | 0,5 ± 0,4^d,e^ | 2,15 ± 0,02^c^ | 1,4 ± 0,3^b,c^ | 0,86 ± 0,01^e^ | |  |
|  | **November** | | 8,76 ± 0,05^a^ | 18 ± 2^b^ | | 1,8 ± 0,2^c^ | 1,79 ± 0,02^d^ | 6,87 ± 0,06^b^ | 10,56 ± 0,02^c^ | |  |
|  | **December** | | 12 ± 2^a^ | 19,74 ± 0,06^b^ | | 8,06 ± 0,02^b^ | 9,23 ± 0,04^b^ | 10,58 ± 0,04^a^ | 10,70 ± 0,08^b^ | |  |
|  | **January 2024** | | 13 ± 3^a^ | 39 ± 10^a^ | | 32,79 ± 0,04^a^ | 67,6 ± 0,2^a^ | 37,4 ± 0,4^a^ | 99,893 ± 0,001^a^ | |  |
|  | **F-values** | | 31,23 | 40,31 | | 20312,77 | 4907,47 | 37,92 | 53077,18 | |  |
|  | **P˃F** | | ˂0,0001 | ˂0,0001 | | ˂0,0001 | ˂0,0001 | ˂0,0001 | ˂0,0001 | |  |
| **NUV6** | **August** | | 0,28 ± 0,01^b^ | | 0,30 ± 0,03^e^ | 1,50 ± 0,06^d^ | 1,97 ± 0,02^d^ | 0,86 ± 0,02^e^ | 1,28 ± 0,08^f^ | |  |
|  | **September** | | 1,37 ± 0,04^a,b^ | 0,42 ± 0,01^d,e^ | | 1,55 ± 0,06^d^ | 1,86 ± 0,05^d^ | 3,07 ± 0,07^d^ | 3,16 ± 0,05^e^ | |  |
|  | **October** | | 2,3 ± 0,6^a,b^ | 0,835 ± 0,007^c,d^ | | 1,043 ± 0,004^e^ | 2,7 ± 0,6^d^ | 5,9 ± 0,4^c^ | 6,2 ± 0,2^d^ | |  |
|  | **November** | | 3 ± 2^a,b^ | 0,682 ± 0,002^c^ | | 2,52 ± 0,06^c^ | 5,2 ± 0,6^c^ | 7,24 ± 0,06^a^ | 32,82 ± 0,01^a^ | |  |
|  | **December** | | 6 ± 4^,b^ | 18,3 ± 0,2^b^ | | 2,75 ± 0,01^b^ | 17 ± 2^b^ | 7,9 ± 0,2^b^ | 8,09 ± 0,06^c^ | |  |
|  | **January 2024** | | 10 ± 5^a^ | 17,1 ± 0,3^a^ | | 14,76 ± 0,06^a^ | 25,6 ± 0,4^a^ | 8,3 ± 0,2^a^ | 20,62 ± 0,02^b^ | |  |
|  | **F-values** | | 3,343 | 1872,92 | | 79357,51 | 414,57 | 1009,36 | 74180,41 | |  |
|  | **P˃F** | | ˂0,0001 | ˂0,0001 | | ˂0,0001 | ˂0,0001 | ˂0,0001 | ˂0,0001 | |  |

Means followed by the same letters in each column are not significantly different according to Tukey’s test at P < 0.05.
