# Supplementary material for: Influence of biosilica treatments and storage receptacles on the quality of maize (Zea mays L.) and common bean (Phaseolus vulgaris L.) seeds during long-term storage
Source: PLoS One. 2026 Mar 11;21(3):e0344033. doi: 10.1371/journal.pone.0344033 (PMC12978491; doi:10.1371/journal.pone.0344033)
Supplement: S5 Table — (DOCX) [file pone.0344033.s005.docx]

**Influence of biosilica treatments and storage receptacles on the quality of maize (*Zea mays* L.) and common bean (*Phaseolus vulgaris* L.) seeds during long-term storage**

Bertrand Zing Zing ^1,2*^, Charles Rostand Mvongo Mvodo ^1^, Valteri Audrey Voula ^1^, Lin Marcellin Messi Ambassa ^1^, Eugene Ejolle Ehabe ^1^, Placide Desiré Belibi Belibi ^3^, Charles Melea Kede ^2^

^1^ Directorate of Scientific Research, Institute of Agricultural Research for Development, P.O. Box 2123, Yaoundé, Cameroon.

^2^ Laboratory of Chemical and Industrial Bioprocess Engineering, National Higher Polytechnic School of Douala, University of Douala, P.O. Box 2701, Douala, Cameroon.

^3^ Department of Inorganic Chemistry, University of Yaoundé I, P.O. Box 812, Yaoundé, Cameroon.

∗ Corresponding author e-mail address: [zingbertrand29@gmail.com](mailto:zingbertrand29@gmail.com) (B.Z.Z)

Bertrand Zing Zing: <https://orcid.org/0000-0002-3892-8950>.

Eugene Ejolle Ehabe: <https://orcid.org/0000-0003-2215-2112>.

Charles Melea Kede: <https://orcid.org/0000-0002-4951-3152>.

**Table 4.** Means weight loss of common beans and maize seeds treated with biosilica after 06 months of storage

| **Variétés** | **Months of storage** |  | |  | **Percent weight loss (%)** | |  |  |  |  |
| --- | --- | --- | --- | --- | --- | --- | --- | --- | --- | --- |
|  |  | **Glass jars (GJ)** | | **Check (GJ)** | **Polypropylene (PP)** | **Check (PP)** | **Polyethylene (PE)** | **Check (PE)** | |  |
| **CMS 8501** | **August** | | 0,4 ± 0,2^b^ | 0,303 ± 0,009^b^ | 0,004± 0,002^e^ | 0,116±0,001^e^ | 0,141± 0,01^a^ | 0,159 ± 0,002^b^ | |  |
|  | **September** | | 0,6 ± 0,3^a,b^ | 0,712 ± 0,03^b^ | 0,19± 0,02^d^ | 0,52± 0,03^c^ | 0,22 ± 0,06^a^ | 0,67 ± 0,07^b^ | |  |
|  | **October** | | 0,7 ± 0,6^a,b^ | 0,9 ± 0,6^a,b^ | 0,32± 0,06^c^ | 0,44± 0,05^d^ | 0,165 ± 0,001^a^ | 1,1 ± 0,7^b^ | |  |
|  | **November** | | 4,56 ± 0,06^a^ | 5 ± 2^a^ | 1,8± 0,8^a^ | 0,822± 0,004^b^ | 0,732 ± 0,002^a^ | 1,3 ± 0,3^b^ | |  |
|  | **December** | | 4,56 ± 0,06^a^ | 5 ± 2^a^ | 0,56± 0,06^b^ | 2,57± 0,03^a^ | 0,44 ± 0,06^a^ | 0,74± 0,11^a^ | |  |
|  | **January 2024** | | / | ^/^ | 1,9± 0,7^a^ | 6,0± 0,2^a^ | 7,2 ± 0,4^a^ | / | |  |
|  | **F-values** | | 6,38 | 11,40 | 26,30 | 5421,45 | 2,85 | 365,29 | |  |
|  | **P˃F** | | 0,008 | 0,001 | ˂0,0001 | ˂0,0001 | 0,081 | ˂0,0001 | |  |
| **CMS 8704** | **August** | | 0,16± 0,08^b^ | 0,29 ± 0,02^a^ | 0,027 ± 0,002^e^ | 0,03 ± 0,01^d^ | 0,229 ± 0,006^a^ | 0,583 ± 0,004^c^ | |  |
|  | **September** | | 0,7 ± 0,4^b^ | 1,4 ± 0,4^a^ | 0,7± 0,3^d^ | 0,74± 0,02^d^ | 1,6 ± 0,2^a^ | 1,14 ± 0,02^b^ | |  |
|  | **October** | | 1,1 ± 0,9^b^ | 0,6 ± 0,3^a^ | 1,92 ± 0,06^c^ | 2,18 ± 0,11^c^ | 1,44 ± 0,06^a^ | 1,6 ± 0,2^a^ | |  |
|  | **November** | | 1,3 ± 0,9^b^ | 1,84 ± 0,56^a^ | 1,88 ± 0,02^c^ | 3,1 ± 0,3^b^ | 2 ± 1^a^ | 1,267 ± 0,005^b^ | |  |
|  | **December** | | 7,2± 0,4^a^ | 8 ± 3^a^ | 5,5 ± 0,5^b^ | 6,5 ± 0,7^a^ | 2 ± 1^a^ | 1,2 ± 0,2^b^ | |  |
|  | **January 2024** | | / | / | 6,4± 0,8^a^ | / | / | / | |  |
|  | **F-values** | | 38,211 | 1,09 | 379,93 | 203,49 | 1,99 | 113,51 | |  |
|  | **P˃F** | | ˂0,0001 | 0,413 | ˂0,0001 | ˂0,0001 | 0,173 | ˂0,0001 | |  |
|  | **August** | | 0,0 ± 0,0^a^ | 0,0 ± 0,0^b^ | 0,04± 0,02^d^ | 0,07± 0,01^d^ | 0,00 ± 0,00^d^ | 0,013 ±0,001^d^ | |  |
| **FEB-190** | **September** | | 0,21 ± 0,02^a^ | 0,059 ± 0,008^a^ | 0,06± 0,03^d^ | 0,082± 0,007^d^ | 0,023 ± 0,002^d^ | 0,026 ± 0,001^d^ | |  |
|  | **October** | | 0,32 ± 0,03^a^ | 0,141 ± 0,009^a^ | 0,04± 0,02^d^ | 0,392± 0,004^c^ | 0,161 ± 0,005^c^ | 0,231 ± 0,002^c^ | |  |
|  | **November** | | 1,35 ± 0,06^a^ | 0,53 ± 0,03^a^ | 0,9± 0,2^c^ | 1,131± 0,003^b^ | 0,83 ± 0,03^a^ | 0,5± 0,2^b^ | |  |
|  | **December** | | 4,52 ± 1,72^a^ | 3,07± 0,05^a^ | 1,532± 0,008^b^ | 4,39± 0,05^a^ | 0,410 ± 0,005^a^ | 0,5± 0,2^b^ | |  |
|  | **January 2024** | | 5,52 ± 2,35^a^ | 18 ± 9^a^ | 2,2± 0,1^a^ | 5,9± 0,5^a^ | 0,28 ± 0,07^a^ | 2,5± 0,3^a^ | |  |
|  | **F-values** | | 0,892 | 6,48 | 337,79 | 76131,98 | 3301,67 | 3929,90 | |  |
|  | **P˃F** | | 0,503 | 0,008 | ˂0,0001 | ˂0,0001 | ˂0,0001 | ˂0,0001 | |  |
| **NUV6** | **August** | | 0,03 ± 0,01^a^ | 0,29 ± 0,03^b^ | 0,300± 0,03^c^ | 0,53±0,01^b^ | 0,066 ± 0,003^d^ | 0,0811± 0,003^d^ | |  |
|  | **September** | | 0,324 ± 0,008^a^ | 0,42 ± 0,21^b^ | 0,3± 0,2^c^ | 0,232± 0,005^b^ | 2,97 ± 0,07^c^ | 2,54 ± 0,02^b^ | |  |
|  | **October** | | 0,6 ± 0,5^a^ | 0,835 ± 0,007^b^ | 0,313± 0,001^c^ | 0,61± 0,08^b^ | 3,1 ± 0,5^c^ | 2,7 ± 0,2^b^ | |  |
|  | **November** | | 0,8 ± 0,4^a^ | 0,682 ± 0,002^b^ | 0,754± 0,011^b^ | 1,19± 0,02^b^ | 7,15 ± 0,06^b^ | 2,2 ± 0,1^c^ | |  |
|  | **December** | | 1,3 ± 0,7^a^ | 18 ± 1^a^ | 0,724± 0,001^b^ | 4,39± 0,9^a^ | 7,8 ± 0,2^a^ | 7,52 ± 0,06^a^ | |  |
|  | **January 2024** | | 2,5± 1,5^a^ | 17 ± 1^a^ | 5,96± 0,04^a^ | 5 ± 1^a^ | 8,9± 0,8^a^ | 4,502± 0,081^a^ | |  |
|  | **F-values** | | 2,79 | 2750,83 | 2046,06 | 49,463 | 753,40 | 2185,38 | |  |
|  | **P˃F** | | 0,087 | ˂0,0001 | ˂0,0001 | ˂0,0001 | ˂0,0001 | ˂0,0001 | |  |

Means followed by the same letters in each column are not significantly different according to Tukey’s test at P < 0.05.
