# Supplementary material for: Influence of biosilica treatments and storage receptacles on the quality of maize (Zea mays L.) and common bean (Phaseolus vulgaris L.) seeds during long-term storage
Source: PLoS One. 2026 Mar 11;21(3):e0344033. doi: 10.1371/journal.pone.0344033 (PMC12978491; doi:10.1371/journal.pone.0344033)
Supplement: S6 Table — (DOCX) [file pone.0344033.s006.docx]

**Influence of biosilica treatments and storage receptacles on the quality of maize (*Zea mays* L.) and common bean (*Phaseolus vulgaris* L.) seeds during long-term storage**

Bertrand Zing Zing ^1,2*^, Charles Rostand Mvongo Mvodo ^1^, Valteri Audrey Voula ^1^, Lin Marcellin Messi Ambassa ^1^, Eugene Ejolle Ehabe ^1^, Placide Desiré Belibi Belibi ^3^, Charles Melea Kede ^2^

^1^ Directorate of Scientific Research, Institute of Agricultural Research for Development, P.O. Box 2123, Yaoundé, Cameroon.

^2^ Laboratory of Chemical and Industrial Bioprocess Engineering, National Higher Polytechnic School of Douala, University of Douala, P.O. Box 2701, Douala, Cameroon.

^3^ Department of Inorganic Chemistry, University of Yaoundé I, P.O. Box 812, Yaoundé, Cameroon.

∗ Corresponding author e-mail address: [zingbertrand29@gmail.com](mailto:zingbertrand29@gmail.com) (B.Z.Z)

Bertrand Zing Zing: <https://orcid.org/0000-0002-3892-8950>.

Eugene Ejolle Ehabe: <https://orcid.org/0000-0003-2215-2112>.

Charles Melea Kede: <https://orcid.org/0000-0002-4951-3152>.

**Table 5.** Means of insect perforation index on treated common beans and maize seeds after 06 months

| **Perforation index (%)** | | | | |
| --- | --- | --- | --- | --- |
|  |  | **Glass jars** |  |  |
| **Months** | **CMS 8501** | **CMS 8704** | **NUV6** | **FEB-190** |
| **August** | 28.63 ± 6.15^b^ | 38.72 ± 7.93^a^ | 20.31 ± 2.06^a^ | 0.00 ± 0.00^b^ |
| **September** | 47.51 ± 3.04^a^ | 40.70 ± 3.47^a^ | 32.12 ± 3.74^a^ | 47.97 ± 16.37^a^ |
| **October** | 46.54 ± 7.93^a^ | 44.28 ± 0.37^a^ | 38.99 ± 5.88^a^ | 49.50 ± 7.99^a^ |
| **November** | 48.32 ± 3.67^a^ | 42.72 ± 6.19^a^ | 31.08 ± 2.02^a^ | 32.29 ± 1.14^a^ |
| **December** | 48.32 ± 3.67^a^ | 44.65 ± 1.25^a^ | 14.39 ± 5.67^a^ | 39.97 ± 3.05^a^ |
| **January 2024** | 50.00 ± 0.01^a^ | 50.00 ± 0.02^a^ | 19.26 ± 8.51^a^ | 25.09 ± 7.75^a.b^ |
| **F-values** | 5.685 | 1.738 | 1.014 | 9.908 |
| **P>F** | 0.006 | 0.200 | 0.451 | 0.001 |
| **Polypropylene bags** | | | | |
| **August** | 50.16 ± 0.09^b^ | 50.00 ± 0.01^a.b^ | 43.28 ± 0.78^a^ | 28.48 ± 0.01^e^ |
| **September** | 50.66 ± 1.86^b^ | 41.78 ± 0.61^c^ | 46.26 ± 0.73^a^ | 42.10 ± 1.46^c^ |
| **October** | 60.53 ± 1.50^a^ | 40.45 ± 1.73^c^ | 28.74 ± 3.92^c^ | 24.27 ± 0.14^f^ |
| **November** | 48.12 ± 3.99^a^ | 44.08 ± 2.81^b.c^ | 33.01 ± 2.10^b.c^ | 50.07 ± 1.82^a^ |
| **December** | 44.87 ± 4.33^a^ | 50.66 ± 1.21^a^ | 13.84 ± 4.33^d^ | 46.58 ± 0.11^b^ |
| **January 2024** | 49.74 ± 0.01^a^ | 48.18 ± 2.58^a.b^ | 36.55 ± 0.28^b^ | 32.68 ± 0.05^d^ |
| **F-values** | 11.760 | 8.248 | 74.223 | 238.590 |
| **P>F** | 0.001 | 0.0001 | ˂0.0001 | ˂0.0001 |
| **Polyethylene bags** | | | | |
| **August** | 16.23 ± 0.00^c^ | 40.78 ± 0.15^d^ | 40.79 ± 0.00^b^ | 0.00 ± 0.00^f^ |
| **September** | 36.77 ± 1.17^a^ | 54.85 ± 0.78^a^ | 49.44 ± 0.42^a^ | 31.64 ± 0.01^d^ |
| **October** | 16.11 ± 0.62^c^ | 47.75 ± 0.75^b.c^ | 48.46 ± 1.09^a^ | 59.44 ± 0.01^a^ |
| **November** | 20.64 ± 0.01^b^ | 41.26 ± 3.11^c.d^ | 78.84 ± 0.28^a^ | 39.29 ± 0.02^c^ |
| **December** | 10.15 ± 2.43^d^ | 41.19 ± 3.59^c.d^ | 49.54 ± 0.42^a^ | 49.68 ± 0.14^b^ |
| **January 2024** | 4.88 ± 0.03^e^ | 50.00 ± 0.02^a.b^ | 28.32 ± 0.02^c^ | 3.58 ± 011^e^ |
| **F-values** | 17.195 | 168.792 | 535.122 | 372913.205 |
| **P>F** | ˂0.0001 | ˂0.0001 | ˂0.0001 | ˂0.0001 |

Means followed by the same letters in each column are not significantly different according to Tukey’s test at P < 0.05.
