# Supplementary material for: Influence of biosilica treatments and storage receptacles on the quality of maize (Zea mays L.) and common bean (Phaseolus vulgaris L.) seeds during long-term storage
Source: PLoS One. 2026 Mar 11;21(3):e0344033. doi: 10.1371/journal.pone.0344033 (PMC12978491; doi:10.1371/journal.pone.0344033)
Supplement: S7 Table — (DOCX) [file pone.0344033.s007.docx]

**Influence of biosilica treatments and storage receptacles on the quality of maize (*Zea mays* L.) and common bean (*Phaseolus vulgaris* L.) seeds during long-term storage**

Bertrand Zing Zing ^1,2*^, Charles Rostand Mvongo Mvodo ^1^, Valteri Audrey Voula ^1^, Lin Marcellin Messi Ambassa ^1^, Eugene Ejolle Ehabe ^1^, Placide Desiré Belibi Belibi ^3^, Charles Melea Kede ^2^

^1^ Directorate of Scientific Research, Institute of Agricultural Research for Development, P.O. Box 2123, Yaoundé, Cameroon.

^2^ Laboratory of Chemical and Industrial Bioprocess Engineering, National Higher Polytechnic School of Douala, University of Douala, P.O. Box 2701, Douala, Cameroon.

^3^ Department of Inorganic Chemistry, University of Yaoundé I, P.O. Box 812, Yaoundé, Cameroon.

∗ Corresponding author e-mail address: [zingbertrand29@gmail.com](mailto:zingbertrand29@gmail.com) (B.Z.Z)

Bertrand Zing Zing: <https://orcid.org/0000-0002-3892-8950>.

Eugene Ejolle Ehabe: <https://orcid.org/0000-0003-2215-2112>.

Charles Melea Kede: <https://orcid.org/0000-0002-4951-3152>.

**Table 6.** Viability of common beans and maize seeds after six 06 months of storage in different materials

| **Cultivars** | **Storage items** |  | | | | |  | **Percentage of viable grains (%)** | | | | |  |  |  |
| --- | --- | --- | --- | --- | --- | --- | --- | --- | --- | --- | --- | --- | --- | --- | --- |
|  |  | **Day 1** | | | **Day 2** | | | **Day 3** | | **Day 4** | **Day 5** | **Day 6** | **Day 7** | |  |
| **CMS 8501** | **Glass jars** | | 0 ± 0^a^ | | 7 ± 4^a^ | | | | 9 ± 4^b^ | 22 ± 9^b^ | 36 ± 9^a^ | 47 ± 11^a^ | 56 ± 9^a^ | |  |
|  | **Polypropylene** | | 0 ± 0^a^ | | 9 ± 4^a^ | | | 27 ± 6^b^ | | 38 ± 4^a^ | 47 ± 6^a^ | 58 ± 14^a^ | 67 ± 11^a^ | |  |
|  | **Polyethylene** | | 0 ± 0^a^ | | 9 ± 4^a^ | | | 18 ± 6^a.b^ | | 22 ± 6^a^ | 53 ± 6^a^ | 67 ± 6^a^ | 80 ± 6^a^ | |  |
|  | **F-values** | | / | | 3 | | | 6.000 | | 1.909 | 3.769 | 1.794 | 16.800 | |  |
|  | **P>F** | | / | | 0.125 | | | 0.037 | | 0.228 | 0.087 | 0.245 | 0.074 | |  |
| **CMS 8704** | **Glass jars** | | 0 ± 0^a^ | | 7 ± 4^a^ | | | | 9± 4^b^ | 29 ± 4^a^ | 31 ± 4^b^ | 36 ± 4^b^ | 40 ± 6^b^ | |  |
|  | **Polypropylene** | | 0 ± 0^a^ | | 9 ± 4^a^ | | | | 27 ± 6^a^ | 38 ± 4^a^ | 47 ± 6^a^ | 51 ± 8^a.b^ | 58 ± 4^a^ | |  |
|  | **Polyethylene** | | 0 ± 0^a^ | | 7 ± 4^a^ | | | | 22 ± 7^a.b^ | 40 ± 6^a^ | 49 ± 4^a^ | 58 ± 4^a^ | 62 ± 4^a^ | |  |
|  | **F-values** | | / | | 3.500 | | | 6.500 | | 14.33 | 11.400 | 8.778 | 4.136 | |  |
|  | **P>F** | | / | | 0.098 | | | 0.031 | | 0.005 | 0.009 | 0.017 | 0.003 | |  |
|  | **Glass jars** | | 0 ± 0^a^ | | 11 ± 4^a^ | | | | 22 ± 4^b^ | 53 ± 11^a^ | 60 ± 6^a^ | 67 ± 6^a^ | 73 ± 5^b^ | |  |
| **FEB-190** | **Polypropylene** | | 0 ± 0^a^ | | 13 ± 6^a^ | | | | 31 ± 4^a^ | 49± 13^a^ | 64 ± 9^a^ | 78 ± 9^a^ | 87 ± 6^a.b^ | |  |
|  | **Polyethylene** | | 0 ± 0^a^ | | 9 ± 4^a^ | | | | 18 ± 6^a^ | 47± 15^a^ | 71 ± 8^a^ | 80 ± 6^a^ | 91 ± 4^a^ | |  |
|  | **F-values** | | | / | | 0.600 | | 4.667 | | 0.143 | 16.636 | 2.385 | 7.429 | |  |
|  | **P>F** | | | / | | 0.579 | | 0.060 | | 0.870 | 0.004 | 0.173 | 0.024 | |  |
| **NUV6** | **Glass jars** | | 0 ± 0^a^ | | 0 ± 0^b^ | | | 9 ± 4^b^ | | 13 ± 0^b^ | 16 ± 4^b^ | 22 ± 4^b^ | 31 ± 4^c^ | |  |
|  | **Polypropylene** | | 0 ± 0^a^ | | 13 ± 3^a^ | | | 18 ± 7^a.b^ | | 31 ± 4^a^ | 44 ± 9^a^ | 49 ± 7^a^ | 51 ± 4^b^ | |  |
|  | **Polyethylene** | | 0 ± 0^a^ | | 9 ± 7^a.b^ | | | 27 ± 6^a^ | | 36 ± 4^a^ | 47 ± 9^a^ | 49 ± 4^a^ | 62 ± 4^a^ | |  |
|  | **F-values** | | / | | 7.400 | | | 6.000 | | 42.000 | 1.118 | 24.00 | 50.333 | |  |
|  | **P>F** | | / | | 0.024 | | | 0.037 | | 0.0001 | 0.387 | 0.001 | 0.0001 | |  |

Means followed by the same letters in each column are not significantly different according to Tukey’s test at P < 0.05.
