# Supplementary material for: Influence of biosilica treatments and storage receptacles on the quality of maize (Zea mays L.) and common bean (Phaseolus vulgaris L.) seeds during long-term storage
Source: PLoS One. 2026 Mar 11;21(3):e0344033. doi: 10.1371/journal.pone.0344033 (PMC12978491; doi:10.1371/journal.pone.0344033)
Supplement: Attachment — Submitted filename: Response to Reviewers.docx [file pone.0344033.s009.docx]

**Effect of stored material using biosilica on grain losses for the eco-conservation of maize (*Zea mays* L.) and common bean (*Phaseolus vulgaris* L.) seeds**

**Journal Requirements:**

1. When submitting your revision, we need you to address these additional requirements.

 Please ensure that your manuscript meets PLOS ONE's style requirements, including those for file naming. The PLOS ONE style templates can be found at

<https://journals.plos.org/plosone/s/file?id=wjVg/PLOSOne_formatting_sample_main_body.pdf> and  <https://journals.plos.org/plosone/s/file?id=ba62/PLOSOne_formatting_sample_title_authors_affiliations.pdf>

 Response: We appreciate your remark dear Editor. The manuscript body has been formatted as PLOS ONE’s style requirements, including those for file naming.

2. Thank you for stating the following financial disclosure:

Royal Society of Chemistry for the Research Fund grant (R24-9789259332)

Please state what role the funders took in the study.  If the funders had no role, please state: "The funders had no role in study design, data collection and analysis, decision to publish, or preparation of the manuscript."

If this statement is not correct, you must amend it as needed.

Please include this amended Role of Funder statement in your cover letter; we will change the online submission form on your behalf.

 Response: Thank you for your remark, dear Editor. The role of the funder has been mentioned as follows in the cover letter: “Royal Society of Chemistry’s Research Fund grant (R24-9789259332) played a crucial role in the collection and analysis of data”.

 3. Please amend the manuscript submission data (via Edit Submission) to include author Dr. Valteri Audrey Voula.

 Response: Many thanks for your remark, dear Editor. The name and surname of Dr. Valteri Audrey Voula have been uploaded to the system.

 4. Please include captions for your Supporting Information files at the end of your manuscript, and update any in-text citations to match accordingly. Please see our Supporting Information guidelines for more information: <http://journals.plos.org/plosone/s/supporting-information>.

 Response: We are so grateful for your remark, dear Editor.

 5. If the reviewer comments include a recommendation to cite specific previously published works, please review and evaluate these publications to determine whether they are relevant and should be cited. There is no requirement to cite these works unless the editor has indicated otherwise.

 Response: Thank you for your remark dear Editor. The comments including recommendation of the Reviewer 2 have been taken in consideration in the manuscript revised”.

**Reviewer #1:** In overall, I think this manuscript didn’t meet high impact journal such as Plos One. It is too simple experiment. and less novelty value. May be, the author should add more treatments which can compare biosilica. Without biosilica as control is too simple analysis. Detail data such as oxygen content, fecundity inset etc. could also be good for this study. Anyway, this paper still good for publication and probably just minor correction for Q2 paper index. The author can refer my comments in main manuscript.

Line 1 and 2: The title should be

Response: Thank you for your remark dear Reviewer 1. The effect of stored material using biosilica on grain losses for eco-conservation of maize (*Zea mays* L.) and common bean (*Phaseolus vulgaris* L.) seeds

Line 78: Please extend the information on biosilica used for store seed treatment. Because it is the main focus of this study. May be a type of biosilica and rate. Or pros and cons of using biosilica as a store seed material.

Response: Many thanks for your remark, dear Reviewer 1. A brief account of Biosilica has been inserted.

Line 96: Be consistent with the order because the results start with maize followed by common bean

Response: We are grateful for your remark, dear Reviewer 1. This was done accordingly.

Line 185 and 186: Repeat information for 100-grain weight

Response: Thank you for your remark, dear Reviewer 1. This was a typing error that is now corrected as 1000-grain weight in the manuscript.

Line 189: No statistical analysis has been conducted for comparing maize and common bean seeds.

Response: We appreciated your comment, dear Reviewer 1. As seeds type have different characteristics and are not infested by the same insect pests, an independent ANOVA followed by a post-hoc test was performed for each crop species. The post-hoc analysis of table 1 (Table 1a and 1b) reveals high significant difference between physical parameters of maize (p = 3.3497E-7) and among common bean seeds (p = 1.6425E-06).

**Table 1**: Layout of post-hoc analysis for maize and common bean cultivars.

| (a) Maize cultivars (CMS 8501: white and CMS 8704: yellow) | | | | | | | | | | | | | |
| --- | --- | --- | --- | --- | --- | --- | --- | --- | --- | --- | --- | --- | --- |
| *Source of variations* | *Sum of Squares* | | *DL* | | | *Mean of squares* | | *F-Value* | | *P>F* | | *F-crit.* | |
| Between groups | 869426.982 | | 5 | | | 173885.396 | | 321.844581 | | 3.3497E-7 | | 4.38737419 | |
| Within groups | 3241.6652 | | 6 | | | 540.277533 | |  | |  | |  | |
| Total | 872668.647 | | 11 | | |  | |  | |  | |  | |
| (b) Common bean cultivars (FEB-190: red and NUV6: black) | | | | | | | | | | | | |  |
| *Source of variations* | *Sum of Squares* | *DL* | | | *Mean of squares* | | *F-Value* | | *P>F* | | *F-crit.* | |  |
| Between groups | 1144176.72 | 5 | | 228835.345 | | | 188.763458 | | 1.6425E-06 | | 4.38737419 | |  |
| Within groups | 7273.7175 | 4 | | 1212.28625 | | |  | |  | |  | |  |
| Total | 1151450.44 | 11 | |  | | |  | |  | |  | |  |

Lines 192-194: About the significant difference of bulk densities for maize and for common bean seeds.

Response: Many thanks for your remark dear Reviewer 1. The sentence was rewritten as bulk densities varied significantly (p ˂ 0.05) between maize cultivars (CMS 8501: 763 ± 13 g/dm^3^; CMS 8704: 714 ± 14 g/dm^3^), but no significant (p > 0.05) variation was observed among common bean cultivars (FEB-190: 784 ± 14 g/dm^3^; NUV6: 810 ± 6 g/dm^3^).

Lines 205-206: Have more information about the significance of storage losses between maize and within common bean seeds

Response: Thank you for this comment, dear Reviewer 1. The Tukey test was performed on all the data related to storage losses and gave the same outputs as the Duncan test. We then performed a post-hoc test to determine the specific differences between cultivars. As shown in Table 2, there was no significant difference (p = 0.563) between maize and common bean seeds, or within them. This suggests that, for each species of maize and common bean, losses increase with storage duration.

**Table 3**: Layout of post-hoc analysis.

| *Source of variations* | *Sum of Squares* | *DL* | *Mean of squares* | *F-Value* | *P>F* | *F-crit* |
| --- | --- | --- | --- | --- | --- | --- |
| Between groups | 1258.91838 | 3 | 419.639461 | 0.696058533 | 0.563530999 | 3.00878657 |
| Within groups | 14469.1094 | 24 | 602.879558 |  |  |  |
| Total | 15728.0278 | 27 |  |  |  |  |

Lines 217-219: The authors should include the nutritional content data or quoted data specific for maize to support this claim.

Response: Thank you for this remark dear Reviewer 1. The quoted nutritional contents for yellow and white maize seeds were appropriately quoted to support our claim.

Line 224: Need quoted paper or previous study to support this claim or just mention further study need to be done.

Response: We appreciate this remark dear Reviewer 1. We mention the need of further studied to better clarify this claim.

Line 228: Based on insect preference testing (yellow) or seed resistant characterization such as soft skin grain.

Response: We are grateful for this remark dear Reviewer 1.

Line 245: I’m facing difficulty to understand how the author presents this result. Unspecific and scattered presentation of results. For me better focus on Maize first and then bean. So, we know which is the best material pun storing maize. Very simple experiment. For me, the best explanation for maize at 5 months of store. PE is the best material for both treated and untreated.

However, for bean store experiment, the damaged pattern was not clear. May be data variation quite high. The author should explain why it wat happen. Overall, PE material was good as well as GJ. But I don’t know why FEB190 show the high damaged for untreated.

Response: Thank you for this remark dear Reviewer 1. For bean store experiment, *A. obtectus* insects can hibernate and multiply inside common bean seeds for some time. The insects are often trapped inside the seeds, which increases the damage of common bean seeds. It is why FEB190 shows the high damage for untreated grains.

Line 294: I think variation or CV value of data were quite high. That the reason why distribution of were not in good pattern. Difficult for to understand. My suggestion, they should convert this data first and then do analysis again. The Mann-Whitney analysis as example.

Response: Thank you for this remark dear Reviewer 1. The Mann-Whitney analysis was done with data transformed (Table 3) and data without transformation (Table 4) for the weight loss.

Table 3. Results of the Mann-Whitney test for the comparison of weight loss with data transformed

| **Cultivars** | **Store materials** | **Mann-Whitney test p value** | **Cultivars** | **Store materials** | **Mann-Whitney test p value** |
| --- | --- | --- | --- | --- | --- |
| Maize | GJs | 0.588 | Common beans | GJs | 0.032* |
|  | GJs Check | 0.996 |  | GJs Check | 0.760 |
|  | PP | 0.755 |  | PP | 0.402 |
|  | PP Check | 0.980 |  | PP Check | 0.862 |
|  | PE | 0.299 |  | PE | 0.261 |
|  | PE Check | 0951 |  | PE Check | 0.830 |

*There was a significant difference between the weight loss results for the compared assays in the control.

Table 4. Results of the Mann-Whitney test for the comparison of weight loss data non-transformed

| **Cultivars** | **Store materials** | **Mann-Whitney test p value** | | **Cultivars** | **Store materials** | | **Mann-Whitney test p value** | |
| --- | --- | --- | --- | --- | --- | --- | --- | --- |
| Maize | GJs | | 0.705 | Common beans | | GJs | | 0.241 |
|  | GJs Check | | 0.767 |  |  | GJs Check | | 0.110 |
|  | PP | | 0.026* |  |  | PP | | 0.367 |
|  | PP Check | | 0.395 |  |  | PP Check | | 0.516 |
|  | PE | | 0.0001** |  |  | PE | | ˂0.0001*** |
|  | PE Check | | 0.335 |  |  | PE Check | | 0.159 |

*There was a significant difference between the weight loss results for the compared assays in the control.

**There was a high significant difference between the weight loss results for the compared assays in the control.

****There was a very high significant difference between the weight loss results for the compared assays in the control.

Line 303: Should have correlation table between weigh loses and insect damaged.

Response: Thank you for this remark dear Reviewer 1. The Pearson’s correlation table between weigh loses and insect damaged of maize (Table 5; page 7) and common bean seeds (table 6; page 7) was done

Lines 347-352: In my opinion, it is better to collect percentage germination for each month store rather than only after 6 months. For me, days after sprouting is not important in this study. Percentage germination after 7 days could be enough

Response: Thank you for this remark, dear Reviewer 1. For our upcoming studied, data on percentage germination will be collected each month and estimated after 07 days.

Line 378: No need to explain again, caused is already in methodology. May be material could be enough

Response: Many thanks for your remark, dear Reviewer 1.

Lines 386-387: The authors should conduct this experiment to confirms this claim.

Response: We appreciate your remark, dear Reviewer 1.

**Reviewer #2:** This study is well designed, has a new innovation. It was successfully laboratory conducted, and was written in good language.

Comments:
Introduction
Line 77 “ to our knowledge……” transfer this sentence to the end of introduction (before: therefore) and include it to clarify the objective of the study
Give a more brief account on Biosilica, its source, previous studies etc…

 Response: We are so grateful for your remark, dear Reviewer 2. Your comments including recommendation have been taken in consideration in the manuscript revised”.

Results and Discussion

Line 313: Please write this sentence “Our findings show have a better outcome than the work of Muhammad et al. [39], which established 61% and 57% damage on red and black landraces of Bambara groundnut (*Vigna subterranean*) seeds, respectively, following attack by *Callosobruchus maculatus,* which translated to weight loss suffered by the affected seeds.” In another manner; for example: “The work of Muhammed et al…….. However, our findings show that…..).

Response: Thank you for your remark, dear Reviewer 2. The whole sentence was rephrased”.

Line 372: “However, no germination was recorded for either common bean seeds or untreated maize grain (negative control) stored for 6 months in GJs, PP and PE bags”. Do you mean: either untreated common beans or maize grains? Thanks

 Response: We appreciated your remark, dear Reviewer 2. Yes. “However, no germination was recorded for either untreated common bean or maize seeds (negative control) stored for 6 months in GJs, PP and PE bags”.

Table 5. Pearson’s correlation between weigh loses and insect damaged of maize seeds

Where, DG_n_ denoted Damaged grains at month n (n=1 to 6) and WL_n_ the Weight loss at month n (n=1 to 6).

Table 6. Pearson’s correlation between weigh loses and insect damaged of common bean seeds
