# Supplementary material for: Influence of biosilica treatments and storage receptacles on the quality of maize (Zea mays L.) and common bean (Phaseolus vulgaris L.) seeds during long-term storage
Source: PLoS One. 2026 Mar 11;21(3):e0344033. doi: 10.1371/journal.pone.0344033 (PMC12978491; doi:10.1371/journal.pone.0344033)
Supplement: Attachment — Submitted filename: evaluation Efficiency of Biosilica on Grain Losses for Eco.docx [file pone.0344033.s010.docx]

**Research evaluation**

**Efficiency of Biosilica on Grain Losses for Eco-conservation of Maize (*Zea mays* L.) and Common Bean (*Phaseolus vulgaris* L.) Seeds**

**Executive Summary**

This manuscript presents an applied and valuable study exploring the use of a rice husk ash derivative, biosilica, for protecting stored grains from pests. The research addresses a problem with significant economic and nutritional implications, especially for small-scale farmers. The manuscript has a solid foundation for publication but requires the resolution of some fundamental gaps to enhance its clarity and compliance with publishing standards.

**Overall Assessment**

Initial Decision: Accept after Major Revisions.

Suitability for the Journal: The research aligns well with the scope of PLOS ONE, presenting experimental work in the field of applied agricultural sciences.

Significance of Contribution: High, as it proposes a practical, potential solution to a global post-harvest problem.

Nature of Feedback: Some points are critical for acceptance, while others aim to improve the quality of analysis and presentation.

**Main Strengths of the Research**

1. Addresses a Relevant Problem: Directly targets the challenge of post-harvest grain loss, which impacts livelihoods and food security.

2. Proposes a Sustainable Alternative: Offers a potential eco-friendly solution by utilizing an agricultural by-product (rice husk) as an alternative to chemical pesticides.

3. Appropriate Experimental Design: Effectively tests the interaction of key variables (container type, grain type, treatment effect) over a realistic storage period.

4. Clear and Actionable Results: Leads to practical recommendations that farmers could implement, including an estimate of the protection period.

**Primary Weaknesses and Development Suggestions**

1. Critical Issue with Data Availability Statement:

Description: A contradiction exists between the submission system response (stating data is in the manuscript) and the dedicated data availability statement in the manuscript (stating it is available upon request). This conflicts with PLOS ONE's publication policy.

Proposed Solution: The authors must upload the core dataset (e.g., counts of damaged grains, weights for each replicate) as Supporting Information files (e.g., an Excel file). The Data Availability Statement must then be updated to accurately reflect this, ensuring consistency across all sections.

2. Methodological Clarity Regarding Treatment Impact:

Description: Adult pests were introduced only at the start, yet the discussion posits the material kills pests. Clarification is needed on how the results are interpreted concerning the pest's full life cycle (including eggs and larvae inside grains).

Proposed Solution: Adding clarification in the methodology or discussion stating that the design measures the cumulative ability of the treatment to suppress pest population development within the semi-closed environment, through its effect on adults and possibly by creating unfavorable conditions for larvae.

3. Improving Results Presentation (Figures):

Description: Many figures (e.g., 2, 3, 4, 5) are overly complex with numerous plot lines, making it difficult for the reader to follow key outcomes.

Proposed Solution: It is suggested to split complex figures into simpler sub-figures (e.g., separating maize from beans, or presenting storage materials separately). Bar charts could help present comparisons more clearly at specific time points.

4. Deepening the Discussion:

Description: The discussion could be enriched by a preliminary mention of the method's potential economic feasibility compared to alternatives, and its expected performance under different humidity or temperature conditions than those in the lab.

Proposed Solution: Adding a short paragraph in the concluding section or discussion touching on these points would enhance the applied value of the research.

**Editorial Notes**

Language: Some minor typographical and grammatical errors require correction (e.g., "obectus" should be corrected to "obtectus").

References: The formatting of some references is incomplete or inconsistent (e.g., references 1, 22, 39). They should be reviewed and standardized according to the journal's style guide.

Clarity: Ensure all abbreviations (GJ, PE, PP) are defined upon first use, and that all figure legends are complete and clear.

**Final Recommendation**

I recommend accepting the manuscript pending Major Revisions. This research has clear applied importance. Resolving the issues related to data policy and improving methodological clarity and presentation will make it a strong, publishable contribution to the literature on sustainable post-harvest management.

**Latakia, 22/12/2025**

**Reviewer**

**Prof. Dr. Nabil ABO KAF**
