# Supplementary material for: Influence of biosilica treatments and storage receptacles on the quality of maize (Zea mays L.) and common bean (Phaseolus vulgaris L.) seeds during long-term storage
Source: PLoS One. 2026 Mar 11;21(3):e0344033. doi: 10.1371/journal.pone.0344033 (PMC12978491; doi:10.1371/journal.pone.0344033)
Supplement: Attachment — Submitted filename: Response_to_Reviewers_auresp_2.docx [file pone.0344033.s011.docx]

**Effect of stored material using biosilica on grain losses for the eco-conservation of maize (*Zea mays* L.) and common bean (*Phaseolus vulgaris* L.) seeds**

**Journal Requirements:**

1. Critical Issue with Data Availability Statement:

Description: A contradiction exists between the submission system response (stating data is in the manuscript) and the dedicated data availability statement in the manuscript (stating it is available upon request). This conflicts with PLOS ONE’s publication policy.

Proposed Solution: The authors must upload the core dataset (e.g., counts of damaged grains, weights for each replicate) as Supporting Information files (e.g., an Excel file). The Data Availability Statement must then be updated to accurately reflect this, ensuring consistency across all sections.

Response: We appreciate your remark, dear Editor. The core dataset, including counts of damaged grains, weights for each replicate, has been uploaded to the system as Supporting Information files. The Data Availability Statement was equally updated.

2. Methodological Clarity Regarding Treatment Impact:

Description: Adult pests were introduced only at the start, yet the discussion posits the material kills pests. Clarification is needed on how the results are interpreted concerning the pest's full life cycle (including eggs and larvae inside grains).

Proposed Solution: Adding clarification in the methodology or discussion stating that the design measures the cumulative ability of the treatment to suppress pest population development within the semi-closed environment, through its effect on adults and possibly by creating unfavorable conditions for larvae.

Response: Thank you for your remark, dear Editor. The clarification proposed was taken into consideration in the revised manuscript”.

3. Improving Results Presentation (Figures):

Description: Many figures (e.g., 2, 3, 4, 5) are overly complex with numerous plot lines, making it difficult for the reader to follow key outcomes.

Proposed Solution: It is suggested to split complex figures into simpler sub-figures (e.g., separating maize from beans, or presenting storage materials separately). Bar charts could help present comparisons more clearly at specific time points.

Response: We appreciate your remark dear Editor. We split complex figures into simpler sub-figures using bar charts to present storage materials separately.

|  |  |
| --- | --- |
|  |  |
| **Fig 2.** Evolution of damaged grains for (a) treated and (b) untreated maize seeds.   | |
|  |  |
|  |  |

**Fig 3.** Evolution of damaged grains for (a) treated and (b) untreated common bean seeds.

|  |  |
| --- | --- |
|  |  |

**Fig 4.** Evolution of weight loss for (a) treated and (b) untreated maize seeds

|  |  |
| --- | --- |
|  |  |

**Fig 5.** Evolution of weight loss for (a) treated and (b) untreated common bean seeds.

|  |  |
| --- | --- |

**Fig 6.** Evolution of perforation index on maize cultivars (a) CMS 8704 and (b) CMS 8501

|  |  |
| --- | --- |

**Fig 7.** Evolution of perforation index on common bean cultivars (a) FEB-190 and (b) NUV6 seeds

|  |  |
| --- | --- |

**Fig 8.** Evolution of viability for maize cultivars (a) CMS 8501 and (b) CMS 8704 seeds

|  |  |
| --- | --- |

**Fig 9.** Evolution of viability for common bean cultivars (a) FEB-190 and (b) NUV6 seeds

4. Deepening the Discussion:

Description: The discussion could be enriched by a preliminary mention of the method’s potential economic feasibility compared to alternatives, and its expected performance under different humidity or temperature conditions than those in the lab.

Proposed Solution: Adding a short paragraph in the concluding section or discussion touching on these points would enhance the applied value of the research.

Response: We are so grateful for your remark, dear Editor.

Editorial Notes

Language: Some minor typographical and grammatical errors require correction (e.g., "obectus" should be corrected to "obtectus").

Response: Thank you for your remark dear Editor. The typographical and grammatical errors were corrected in the revised manuscript.

References: The formatting of some references is incomplete or inconsistent (e.g., references 1, 22, 39). They should be reviewed and standardized according to the journal’s style guide.

Response: Many thanks for your remark, dear Editor. The references 1, 22 and 39 were formatted and standardized according to the journal’s style guide.

Clarity: Ensure all abbreviations (GJ, PE, PP) are defined upon first use, and that all figure legends are complete and clear.

Response: We are so grateful for your remark, dear Editor.
