# Supplementary material for: Influence of biosilica treatments and storage receptacles on the quality of maize (Zea mays L.) and common bean (Phaseolus vulgaris L.) seeds during long-term storage
Source: PLoS One. 2026 Mar 11;21(3):e0344033. doi: 10.1371/journal.pone.0344033 (PMC12978491; doi:10.1371/journal.pone.0344033)
Supplement: Attachment — Submitted filename: PONE-D-25-33584-Reviewer comments.pdf [file pone.0344033.s008.pdf]

PLOS ONE

# Efficiency of Biosilica on Grain Losses for Eco-conservation of Maize (Zea mays L.) and Common Bean (Phaseolus vulgaris L.) Seeds

--Manuscript Draft--

| | |
|---|---|
| **Manuscript Number:** | PONE-D-25-33584 |
| **Article Type:** | Research Article |
| **Full Title:** | Efficiency of Biosilica on Grain Losses for Eco-conservation of Maize (Zea mays L.) and Common Bean (Phaseolus vulgaris L.) Seeds |
| **Short Title:** | Eco-conservation of Maize (Zea mays L.) and Common Bean (Phaseolus vulgaris L.) Seeds with Biosilica |
| **Corresponding Author:** | Zing Zing Bertrand, M.D<br>Institute of Agricultural Research for Development: Institut de recherche agricole pour le developpement<br>YAOUNDE, CENTRE CAMEROON |
| **Keywords:** | Biosilica; Eco-conservation; Grain losses; Phaseolus vulgaris; Viability; Zea mays. |
| **Abstract:** | Common bean (Phaseolus vulgaris L.) is a major source of affordable, high-quality dietary protein that complements maize (Zea mays L.)-based diets. Traditional storage of these seeds in sacks and glass jars without treatment often leads to infestation by insect pests, such as Acanthoscelides obtectus and Sitophilus zeamais, causing significant grain losses. This study evaluated grain losses, specifically grain damage and weight loss, after mixing biosilica with maize and common bean seeds, which were stored in glass jars (GJs), polyethylene (PE), and polypropylene (PP) bags, alongside negative controls (without biosilica), over a six-month period. Seed viability was also assessed during the storage period. The initial moisture level of less than 13% for all grain cultivars species was favorable for storage. After six months of storage without treatment, less than 50% of common beans (FEB 190 and NUV6) and more than 65% of maize seeds (CMS 8501 and CMS 8704) were affected. For treated seeds stored using different materials, only PE bags and GJs were more effective for common beans than for maize seeds after five months of storage. However, by the sixth month, all three conservation materials showed an insect perforation index of less than 50%, suggesting a positive protective effect of the biosilica. With seed viability exceeding 80% for both maize and common beans treated and stored in PE and PP over six months, the application of biosilica-coated seeds in bags can reduce storage losses. This, in turn, can help farmers improve safe grain storage over longer periods, ultimately enhancing their livelihoods and income. |
| **Order of Authors:** | Zing Zing Bertrand, M.D |
| | Charles Rostand Mvongo Mvodo, PhD |
| | Lin Marcellin Messi Ambassa, PhD |
| | Eugene Ejolle of Agricultural Research for Development Ehabe, PhD |
| | Placide Désiré Belibi Belibi, PhD |
| | Charles Melea Kede, PhD |
| **Additional Information:** | |
| **Question** | **Response** |
| **Financial Disclosure**<br><br>Enter a financial disclosure statement that describes the sources of funding for the work included in this submission. Review the submission guidelines for detailed requirements. View published research | Yes |

Powered by Editorial Manager® and ProduXion Manager® from Aries Systems Corporation

| | |
|---|---|
| articles from *PLOS ONE* for specific examples.<br><br>This statement is required for submission and **will appear in the published article** if the submission is accepted. Please make sure it is accurate.<br><br>**Funded studies**<br>Enter a statement with the following details:<br>• Initials of the authors who received each award<br>• Grant numbers awarded to each author<br>• The full name of each funder<br>• URL of each funder website<br>• Did the sponsors or funders play any role in the study design, data collection and analysis, decision to publish, or preparation of the manuscript?<br><br>Did you receive funding for this work? | |
| Please add funding details.<br>as follow-up to "**Financial Disclosure**<br><br>Enter a financial disclosure statement that describes the sources of funding for the work included in this submission. Review the submission guidelines for detailed requirements. View published research articles from *PLOS ONE* for specific examples.<br><br>This statement is required for submission and **will appear in the published article** if the submission is accepted. Please make sure it is accurate. | Royal Society of Chemistry for the Research Fund grant (R24-9789259332) |

| | |
|---|---|
| **Funded studies**<br>Enter a statement with the following details:<br>• Initials of the authors who received each award<br>• Grant numbers awarded to each author<br>• The full name of each funder<br>• URL of each funder website<br>• Did the sponsors or funders play any role in the study design, data collection and analysis, decision to publish, or preparation of the manuscript?<br><br>Did you receive funding for this work?" | |
| Please select the country of your main research funder (please select carefully as in some cases this is used in fee calculation).<br>as follow-up to "**Financial Disclosure**<br><br>Enter a financial disclosure statement that describes the sources of funding for the work included in this submission. Review the submission guidelines for detailed requirements. View published research articles from *PLOS ONE* for specific examples.<br><br>This statement is required for submission and **will appear in the published article** if the submission is accepted. Please make sure it is accurate.<br><br>**Funded studies**<br>Enter a statement with the following details:<br>• Initials of the authors who received each award<br>• Grant numbers awarded to each author<br>• The full name of each funder<br>• URL of each funder website<br>• Did the sponsors or funders play any role in the study design, data collection and analysis, decision to publish, or preparation of the manuscript?<br><br>Did you receive funding for this work?" | UNITED KINGDOM - GB |
| **Competing Interests** | The authors have declared that no competing interests exist. |

*Powered by Editorial Manager® and ProduXion Manager® from Aries Systems Corporation*

| | |
|---|---|
| Use the instructions below to enter a competing interest statement for this submission. On behalf of all authors, disclose any competing interests that could be perceived to bias this work—acknowledging all financial support and any other relevant financial or non-financial competing interests.<br><br>This statement is required for submission and **will appear in the published article** if the submission is accepted. Please make sure it is accurate and that any funding sources listed in your Funding Information later in the submission form are also declared in your Financial Disclosure statement.<br><br>View published research articles from *PLOS ONE* for specific examples.<br><br>**NO authors have competing interests**<br><br>Enter: *The authors have declared that no competing interests exist.*<br><br>**Authors with competing interests**<br><br>Enter competing interest details beginning with this statement:<br><br>*I have read the journal's policy and the authors of this manuscript have the following competing interests: [insert competing interests here]*<br><br>* typeset | |
| **Ethics Statement**<br><br>Enter an ethics statement for this submission. This statement is required if the study involved:<br><br>• Human participants<br>• Human specimens or tissue<br>• Vertebrate animals or cephalopods<br>• Vertebrate embryos or tissues | "N/A" |

| | |
|---|---|
| • Field research<br><br>Write "N/A" if the submission does not require an ethics statement.<br><br>General guidance is provided below. Consult the submission guidelines for detailed instructions. **Make sure that all information entered here is included in the Methods section of the manuscript.** | |

| | |
|---|---|
| **Format for specific study types**<br><br>**Human Subject Research (involving human participants and/or tissue)**<br>• Give the name of the institutional review board or ethics committee that approved the study<br>• Include the approval number and/or a statement indicating approval of this research<br>• Indicate the form of consent obtained (written/oral) or the reason that consent was not obtained (e.g. the data were analyzed anonymously)<br><br>**Animal Research (involving vertebrate animals, embryos or tissues)**<br>• Provide the name of the Institutional Animal Care and Use Committee (IACUC) or other relevant ethics board that reviewed the study protocol, and indicate whether they approved this research or granted a formal waiver of ethical approval<br>• Include an approval number if one was obtained<br>• If the study involved *non-human primates*, add *additional details* about animal welfare and steps taken to ameliorate suffering<br>• If anesthesia, euthanasia, or any kind of animal sacrifice is part of the study, include briefly which substances and/or methods were applied<br><br>**Field Research**<br><br>Include the following details if this study involves the collection of plant, animal, or other materials from a natural setting:<br>• Field permit number<br>• Name of the institution or relevant body that granted permission | |
| **Data Availability**<br><br>Authors are required to make all data underlying the findings described fully available, without restriction, and from the time of publication. PLOS allows rare exceptions to address legal and ethical concerns. See the PLOS Data Policy and FAQ for detailed information. | Yes - all data are fully available without restriction |

| | |
|---|---|
| A Data Availability Statement describing where the data can be found is required at submission. Your answers to this question constitute the Data Availability Statement and **will be published in the article**, if accepted.<br><br>**Important:** Stating 'data available on request from the author' is not sufficient. If your data are only available upon request, select 'No' for the first question and explain your exceptional situation in the text box.<br><br>Do the authors confirm that all data underlying the findings described in their manuscript are fully available without restriction? | |
| **Describe where the data may be found in full sentences. If you are copying our sample text, replace any instances of XXX with the appropriate details.**<br><br>• If the data are **held or will be held in a public repository**, include URLs, accession numbers or DOIs. If this information will only be available after acceptance, indicate this by ticking the box below. For example: *All XXX files are available from the XXX database (accession number(s) XXX, XXX.*).<br>• If the data are all contained **within the manuscript and/or Supporting Information files**, enter the following: *All relevant data are within the manuscript and its Supporting Information files.*<br>• If neither of these applies but you are able to provide **details of access elsewhere**, with or without limitations, please do so. For example:<br><br>*Data cannot be shared publicly because of [XXX]. Data are available from the XXX Institutional Data Access / Ethics Committee (contact via XXX) for researchers who meet the criteria for access to confidential data.*<br><br>*The data underlying the results presented in the study are available from (include the name of the third party* | All relevant data are within the manuscript and its Supporting Information files |

| | |
|---|---|
| *and contact information or URL).*<br>• This text is appropriate if the data are owned by a third party and authors do not have permission to share the data.<br><br>* typeset | |
| Additional data availability information: | |

Manuscript Click here to access/download;Manuscript;Manuscript.docx

# Efficiency of Biosilica on Grain Losses for Eco-conservation of Maize (*Zea mays* L.) and Common Bean (*Phaseolus vulgaris* L.) Seeds

Bertrand Zing Zing [1,2*], Charles Rostand Mvongo Mvodo [1], Valteri Audrey Voula [1], Lin Marcellin Messi Ambassa [1], Eugene Ejolle Ehabe [1], Placide Desiré Belibi Belibi [3], Charles Melea Kede [2]

[1] Directorate of Scientific Research, Institute of Agricultural Research for Development, P.O. Box 2123, Yaoundé, Cameroon.

[2] Laboratory of Chemical and Industrial Bioprocess Engineering, National Higher Polytechnic School of Douala, University of Douala, P.O. Box 2701, Douala, Cameroon.

[3] Department of Inorganic Chemistry, University of Yaoundé I, P.O. Box 812, Yaoundé, Cameroon.

∗ Corresponding author e-mail address: zingbertrand29@gmail.com (B.Z.Z)

Bertrand Zing Zing: https://orcid.org/0000-0002-3892-8950.

Eugene Ejolle Ehabe: https://orcid.org/0000-0003-2215-2112.

Charles Melea Kede: https://orcid.org/0000-0002-4951-3152.

## Abstract

Common bean (*Phaseolus vulgaris* L.) is a major source of affordable, high-quality dietary protein that complements maize (*Zea mays* L.)-based diets. Traditional storage of these seeds in sacks and glass jars without treatment often leads to infestation by insect pests, such as *Acanthoscelides obtectus* and *Sitophilus zeamais,* causing significant grain losses. This study evaluated grain losses, specifically grain damage and weight loss, after mixing biosilica with maize and common bean seeds, which were stored in glass jars (GJs), polyethylene (PE), and polypropylene (PP) bags, alongside negative controls (without biosilica), over a six-month period. Seed viability was also assessed during the storage period. The initial moisture level of less than 13% for all grain cultivars species was favorable for storage. After six months of storage without treatment, less than 50% of common beans (FEB 190 and NUV6) and more than 65% of maize seeds (CMS 8501 and CMS 8704) were affected. For treated seeds stored using different materials, only PE bags and GJs were more effective for common beans than for maize seeds after five months of storage. However, by the sixth month, all three conservation materials showed an insect perforation index of less than 50%, suggesting a

positive protective effect of the biosilica. With seed viability exceeding 80% for both maize and common beans treated and stored in PE and PP over six months, the application of biosilica-coated seeds in bags can reduce storage losses. This, in turn, can help farmers improve safe grain storage over longer periods, ultimately enhancing their livelihoods and income.

**Keywords:** Biosilica, Eco-conservation, Grain losses, *Phaseolus vulgaris*, Viability, *Zea mays*.

## Article Highlights

- Small-scale farmers in Cameroon use traditional storage, which may result in significant grain losses.
- Polyethylene and polypropylene bags better protect common bean seeds than maize seeds against damage by insect pests.
- Biosilica mixed with grains extends storability and maintains grain viability above 80% after 6 months.

## 1. Introduction

Maize (*Zea mays* L.) and common bean (*Phaseolus vulgaris* L.) are vital dietary component, playing a crucial role in food security, income generation, and livelihoods worldwide [1]. The common bean is an important source of affordable protein that complements maize-based diets, both of which are consumed by approximately 2.5% of the global population [2]. Both grains are highly seasonal in production, and once harvested, farmers and processors must preserve them to ensure stable year-round consumption for their families, protect against market price fluctuations, and support planting [3]. In Cameroon, these grains are typically stored on a small scale in jute or woven polypropylene sacks, which are easy to handle, allow air circulation when properly placed, and do not require special storage spaces.

However, these sacks offer limited durability and inadequate protection against humidity and insect pests like *Sitophilus zeamais* and *Acanthoscelides obtectus* [4]. Depending on storage conditions and the type of sacks used, *A. obectus* can cause seed losses ranging from 48% to 100%, while, *S. zeamais* can damage up to 90% of unprotected maize grains in storage [5, 6]. These economically important pests bore into the kernels, feed on undamaged grains, and selectively consume nutritive components of stored maize and common bean seeds [7-9]. In addition, by feeding on whole or broken grains, these insect pests decrease the mass and/or

volume, reduce physiological quality and seed viability, and thereby contribute significantly to food insecurity and economic strain, particularly in Sub-Saharan Africa [1, 10, 11].

To maintain the health of stored seeds, grains are stored in Purdue Improved Cowpea Storage bags [12, 13], improved hermetic bags like GrainPro Super Bags, AgroZ® Plus, ZeroFly® bags, and triple layer bags [14, 15]. Although these hermetic bags are effective, their high cost makes them less appealing to farmers in many regions of Sub-Saharan Africa. As a result, chemical pesticides such as deltamethrine and pyrimiphos-methyl are often mixed with seeds before storage in sacks. While effective, these chemicals pose environmental and health risks due to residual pesticides on seeds, prompting researchers to explore sustainable alternatives [16-18]. Research has demonstrated the effectiveness of plant volatile compounds and essential oils against these insect pests. However, their short shelf life due to volatility and evaporation under heat, light, and moisture limits their wide use [19-21]. To our knowledge, studies evaluating the efficiency of manually mixed biosilica with infested maize and common bean seeds stored in glass jars (GJs), polypropylene (PP) and polyethylene (PE) bags to assess real losses under small-farm conditions over six months are scarce.

In our earlier work, we reported significant insecticidal activity through contact-ingestion of biosilica by *A. obtectus* and *S. zeamais*, insect pests of common bean and maize seeds [1]. The use of biosilica as a bioinsecticide is attributed to the presence of silanol and siloxane functional groups, with the hydrophilic -OH group of silanol capable of binding to the insect's cuticle, leading to death by dehydration. Additionally, we hypothesize that the reactivity of silanol and siloxane functional groups mitigates moisture from seed evaporation and contributes to the dehydration the insect pests, thereby ensuring the sustainable and safe storability of grains over a prolonged period.

Therefore, the objective of this study is to evaluate storage parameters: namely grain damage, weight loss, and insect perforation index, of infested maize and common bean grains mixed with biosilica during six months of storage in GJs, PP and PE bags for six months at a laboratory scale.

## 2. Methodology

### 2.1. Collection of grain cultivars species, insect rearing, and biosilica obtention

Common beans (FEB 190 and NUV6 cultivars) and maize (CMS 8501 and CMS 8704 cultivars) used for the experiment were procured in June 2023 from the Multipurpose Station of IRAD Foumbot and the Division of Crop Production IRAD, Yaoundé (Cameroon), located in the West

and Centre regions, respectively. The insects used for bioassay tests were reared as described by Zing et al. [1]. Typically, *A. obtectus* and *S. zeamais*–infested common beans (cultivars FEB-192 and NUV6) and maize (cultivars CMS 8501 and CMS 8704) were reared from April to July 2023 and February to May 2023, respectively, in the Entomology Laboratory of IRAD Yaoundé (Cameroon). Adult insects were placed in 1-L glass jars with lid mesh (2 mm) containing 250 g of each grain cultivar and stored for 60 days under controlled conditions (in the dark at 28 ± 2°C and 70 ± 10% relative humidity). The procedure for obtaining the biosilica and its characterization were outlined formerly by Zing et al. [1].

### 2.2. Physical parameters of maize and common bean seeds

This was assessed in triplicate to determine moisture content, 100- and 1000-grain weight, bulk density, graining, and the average grain weight of each variety of maize and common beans. The moisture content of each seed was determined using a calibrated moisture meter (Dickey-john Corp. Auburn, IL 62615 USA). The 100- and 1000-grain weights were determined by weighing the corresponding masses for each seed cultivar. Graining was defined as the number of seeds required to reach a weight of 100 g. The average grain weight was calculated as the ratio of the weight of 100 grains to 100. Bulk density was determined as the ratio of the mass of the sample (g) to its volume (mL).

### 2.3. Storage loss estimation

Masses of 500 ± 0.6 g of the maize (CMS 8704 and CMS 8501 cultivars) and common bean (NUV6 and FEB 190 cultivars) seeds were weighed and placed in 1200 mL glass jars. Fifty pairs of insects were then added. The glass jars were covered with muslin cloth to allow for aeration while preventing the insects from escaping. The jars were kept at room temperature in the laboratory for six months. For each triplicate, the difference in grain quantity at the beginning of the storage period ($Y_0$) and the end ($Y_t$), expressed as a percentage, was determined monthly over the six-month period (Eq.1).

$$\%\ \text{Storage loss} = \frac{(Y_0 - Y_t)}{Y_0} \times 100 \qquad (1)$$

### 2.4. Grain damage, weight loss, and insect perforation index in storage items

Grain samples were stored in three types of materials, namely: woven PP bags (SPP 60 x 100, white), PE bags (high-density, non-toxic, odorless, with 80% to 90% crystallinity, a softening point of 125 to 135°C, and an operating temperature of up to 100°C), and GJs procured from the central market in Yaoundé city. Maize and common bean seeds (200 g each) were manually

well mixed with 10 g of biosilica in PE and PP bags separately. Then, 50 g of each seed type was put in the glass jars separately and manually well mixed for 5 min with 2.25 g of biosilica powders, respectively, to ensure even distribution within the grains. Fifty and ten pairs of adult insects were introduced in both the bags and GJs, respectively, and the containers were tightly covered. All the experiments were repeated in triplicate. Grain damage, weight loss, and the insect perforation index (IPI) were assessed monthly for up to 6 months, along with grain viability in the final month.

Insect-damaged grains were assessed by the count method, where seeds from different maize and common bean samples were individually inspected for the presence of holes. The number of damaged and undamaged grains was recorded, and the percentage of damaged grains (Eq. 2) was calculated as the ratio of damaged grains to the total number of grains (damaged + undamaged) in each replicate. Grain weight loss was calculated (Eq. 3) using the count and weight method developed by Misele et al. [22], and the IPI was calculated (Eq. 4) as described by Gever and Echezona [23]. All experiments were performed under controlled laboratory conditions of temperature (T = 24.1-25.6 °C) and relative humidity (RH = 70.7-74.5 %).

$$\%\ \text{Damaged grain} = \frac{N_d}{(N_d + N_u)} \times 100 \qquad (2)$$

$$\%\ \text{Weight loss} = \frac{((W_u * N_d) - (w_d * N_u))}{W_u(N_d + N_u)} \times 100 \qquad (3)$$

$$\text{IPI} = \frac{G_{tg}}{(G_{ptc} + G_{tg})} \times 100 \qquad (4)$$

Where $W_u$ = weight of undamaged grains, $N_d$ = number of damaged grains, $W_d$ = weight of damaged grains, $N_u$ = number of undamaged grains, $G_{tg}$ = percentage of treated grains and $G_{ptc}$ = percentage of perforated grains in the control.

### 2.5. Determination of viability of treated grains in different materials

The viability of treated seeds was assessed using the method described by Berhe et al. [24] with slight modification. Typically, 15 seeds of maize and common beans were randomly sampled from the GJs, PP, and PE bags and placed in sterilized Petri dishes containing a moistened cotton layer. The Petri dishes were then randomly organized, on a platform in the laboratory, and the seeds were moistened daily. After 7 days, germinated grains were counted, and the percentage of germinated seed calculated using equation 5.

$$\text{Germination} = \frac{G_{ss}}{G_{ig}} \times 100 \qquad (5)$$

Where $G_{ss}$ = total number of sprouted seed and $G_{ig}$ = number of initial grains.

### 2.6. Data Analysis

Data entered in an Excel spreadsheet were analyzed using XLSTAT Version 2016.02.27444 software. For all parameters investigated, non-linear and linear graphs were plotted using GraphPad Prism Version 9.5.1 after performing a one-way ANOVA to separate significant means using Tukey's test. The post-hoc test was used to determine specific differences between cultivar whereas, an independent t-test was applied to compare the means between the two grain species at 5% probability level and 95% confidence limits. The significance of a parameter was determined at a 95% confidence interval (significant for $p < 0.05$; non-significant for $p > 0.05$).

## 3. Results and discussion

### 3.1. Physical parameters of common bean and maize seeds

The independent t-test results presented in Table 1 reveal significant differences ($P < 0.05$) between the means of graining, mass of 100 grains, mass of 1000 grains, bulk density, and moisture content of maize. This highlights substantial variation in the physical characteristics of maize seeds. In contrast, no significant differences ($P > 0.05$) were observed in the means of average mass, bulk densities, or moisture contents for common bean species. The higher graining obtained in common beans NUV6 suggests that more grains are required to form a homogeneous 100 g sample. The average mass and mass of 1000 grains of maize decrease with decreasing grain size, whereas in common beans NUV6, these values increase with grain size.

A significant difference ($P < 0.05$) was also found in the 100-grain weight between white maize CMS 8501 (25.9 ± 0.5g) and yellow maize CMS 8704 (22.7 ± 0.6g), with white maize kernels weighs approximately 1.14-times more than yellow maize kernels. However, no significant difference ($P > 0.05$) was observed in the 100-grain weight between common bean cultivars FEB 190 (18.3 ± 0.5g) and NUV6 (17.8 ± 0.6g). According to the size classification of Kläsener et al. [25], all evaluated bean samples have medium-sized grains (< 40 g 100 grains$^{-1}$). Another significant difference ($P < 0.05$) was observed in the 100-grain weight of maize seeds, with CMS 8501 having the highest value. No such difference was found among common bean seeds. Moisture contents of all kernels were below 13% and did not differ significantly ($P > 0.05$) between maize cultivars (CMS 8704 and CMS 8501) or between common bean cultivars (FEB 190 and NUV6). However, a significant difference ($P < 0.05$) was observed between maize and

common bean kernels. Moisture contents below 13% indicate that they are suitable for storage and would limit the development and proliferation of pathogenic grain fungi [26].

Bulk densities varied significantly ($P < 0.05$) between maize and common bean seeds, and also among maize cultivars (CMS 8501: 763 ± 13 g/dm$^3$; CMS 8704: 714 ± 14 g/dm$^3$) and common bean cultivars (FEB 190: 784 ± 14 g/dm$^3$; NUV6: 810 ± 6 g/dm$^3$). This parameter is used to classify grains for trading and processing into food products [27]. In this study, the moisture content of maize CMS 8501 increased with higher bulk density, whereas it decreased with higher bulk density in CMS 8704 increases. Conversely, the bulk density of both common bean cultivars decreased as their moisture contents decreased. The determination of these parameters is essential during trading to ensure fair pricing for both farmers and seed companies.

**Table 1.** Physical compositions of common beans (FEB 190 and NUV6) and maize (CMS 8501 and CMS 8704) raw grains.

| **Grain species** | **Graining** | **Average mass (g)** | **Mass of 100 grains (g)** | **Mass of 1000 grains (g)** | **Bulk density (g/dm$^3$)** | **Moisture contents (%)** |
|---|---|---|---|---|---|---|
| CMS 8501 | 387 ± 7$^b$ | 0.25 ± 0.07$^a$ | 25.9 ± 0.5$^a$ | 261 ± 4$^a$ | 763 ± 13$^a$ | 12.3 ± 0.4$^a$ |
| CMS 8704 | 441 ± 7$^a$ | 0.23 ± 0.04$^a$ | 22.7 ± 0.6$^b$ | 227 ± 5$^b$ | 714 ± 14$^b$ | 12.0 ± 0.6$^b$ |
| $P (T \leq t)$ | 0.0001 | 0.073 | 0.0001 | 0.001 | 0.0004 | 0.033 |
| NUV6 | 604 ± 16$^a$ | 0.18 ± 0.06$^a$ | 33.8 ± 0.6$^a$ | 347 ± 12$^a$ | 810 ± 6$^a$ | 12.45 ± 0.06$^a$ |
| FEB 190 | 563 ± 15$^b$ | 0.19 ± 0.01$^a$ | 24.3 ± 0.5$^b$ | 237 ± 6$^b$ | 784 ± 14$^a$ | 12.88 ± 0.07$^a$ |
| $P (T \leq t)$ | 0.002 | 0.423 | 0.001 | 0.002 | 0.112 | 0.298 |

Means followed by the same letters in each column are not significantly different according to the independent t-test at $P < 0.05$.

### 3.2. Storage losses of common beans and maize seeds

Fig 1 shows an increase in losses of common bean and maize grains to which insects were added at trial set-up for 6 months.

**Fig 1**. Evolution of storage losses for (a) maize and (b) common beans cultivars during 6 months on glass jars in the laboratory.

The post-hoc analysis of data plotted in Fig 1 shows no significant differences ($F = 0.70$; $DF = 3$; $P = 0.56$) in losses between maize and common bean cultivars. This suggests that, for each maize and common bean species, mean losses increase with storage duration. From Fig 1a, maize seeds (CMS 8704 and CMS 8501) suffered more than 50% loss, with the greatest loss being 85% for CMS 8704 and 65% for CMS 8501 of kernels initially seeded with

the same number of *S. zeamais* during the 6th month of storage. The higher loss of CMS 8704 during the 6 months of storage is likely related to its higher carbohydrate content in the form of starch, sugar, cellulose, and non-cellulosic polysaccharides in the pericarp compared to CMS 8501 [28]. Another factor that lower grains losses of CMS 8501 compared to CMS 8704 coulb be the white color of the grains. In fact, Cortese et al. [29] have already demonstrated that the brightness of the white color in maize grains possibly has a repellent effect on adults of *S. zeamais* known as antixenosis effect. In contrast, common bean seeds (NUV6 and FEB 190, Fig 1b) showed less than 50% loss over 6 months, possibly due to their smaller average weight, which makes it harder for *A. obtectus* to perforate them compared to maize kernels. The increased storage losses in maize grains may be attributed to the hardness of their kernels and the long lifespan (3-6 months or more) of *S. zeamais* adults, which take longer to bore into kernels, causing significant damage to both grains, with yellow maize being more susceptible [30]. The robust mandibular structure of *S. zeamais* enables its mouthparts to attack compact grains [31]. This study confirms that storage losses of maize seeds were higher than those of common beans from the second to the sixth month of storage, highlighting the severe infestation by *S. zeamais* and *A. obtectus,* which destroy seeds and contribute to food insecurity and economic strain.

### 3.3. Effect of biosilica on grain damage for maize and common beans under storage

The evolution of damaged grains in stored items treated with biosilica powder and controls (without biosilica) are shown in Fig 2.

**Fig 2**. Evolution of damaged grains for (a) treated maize, (b) untreated maize, (c) treated common bean and (d) untreated common bean seeds.

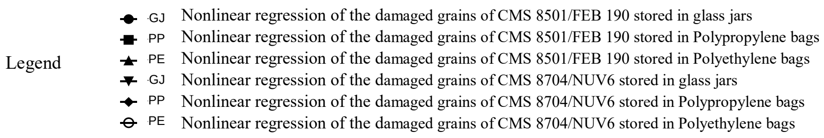


From Figs 2a and 2b, seeds treated and stored in GJs showed a rapid increase in damage, from 1.2% to 100% for CMS 8501 and from 0.8% to 100% for CMS 8704. In contrast, common beans treated and stored under the same conditions exhibited an increase in damage from 1.37% to 13% for FEB 190 and from 0.28% to 10% for NUV6 (Fig. 2c and 2d). For untreated grains stored in GJs, the percentage of damaged grains reached 100% after 6 months for both maize

and common beans. This difference in the percentage of damaged kernels may be due to the difficulty *A. obtectus* insects face in moving between the kernels and perforate them. The high percentage of damaged maize and common bean seeds in GJs may result from oxygen diffusion through the fabric openings, which reduces the insecticidal activity of the biosilica. The highest grain damage observed in PP bags during the 6-month storage period likely resulted from decreased insecticidal activity of biosilica, possibly due to ambient oxygen entering the bags and supporting insect respiration, as well as temperature fluctuations between the interior and exterior of the bags [32]. The slight difference between treated and untreated seeds stored in PP bags is attributed to the activity of biosilica, which when mixed with grains, induces insect inappetence compared to control grains, which suffer heavy damage.

For treated seeds stored in woven PP bags, damage varied from 0.19% to 32.80% (FEB 190), 0.44% to 89.45% (CMS 8501), and 0.51% to 93% (CMS 8704) from the first to the sixth month, respectively. For NUV6 grains, the percentage of damage slightly increased from 1.50% to 1.55%) in the first two months, decreased to 1.04% in the third month, and then increased again from 2.52% to 14.76% from the fourth to the sixth month. For untreated grains stored in PP bags, damage reached 25.6% and 67.6% for common beans (NUV6 and FEB 190, respectively) and 100% for maize (CMS 8704 and CMS 8501) after 6 months. This grain damage by *A. obtectus* and *S. zeamais* on common beans and maize may be associated with the porous structure of PP bags, which allows oxygen availability for insect survival and reduces the insecticidal activity of biosilica. The percentages of damaged NUV6 grains in this study are lower than the values of 17% and 30% reported by Kuyu et al. [33] when evaluating different grain storage technologies against insect pests.

For seeds treated and stored in PE bags, and untreated FEB 190 grains, less than 3% and 100% damaged, respectively, were observed at the first and sixth months. However, after 6 months, 100% of CMS 8704 and CMS 8501 grains were damaged, compared to 20.62% and 99.89% for NUV6 and FEB 190. The lower grain damage observed in PE bags, compared to GJs and PP bags, could be due to the absence of holes or pores, which prevents oxygen penetration for insect respiration without reducing the insecticidal activity of the biosilica. The significant increase in grain damage between the 5$^{th}$ and 6$^{th}$ month across storage types is likely because grains were mixed with biosilica monthly until the third month and stored in the different items. From the 4$^{th}$ to the 6$^{th}$ month, no further biosilica was added or replaced. This was done to asssess the stability of biosilica in reducing grain damage in GJs, PP, and PE bags over 3 months

of storage. Consequently, over 50% of grains were damaged after the 6th month across all storage types and their controls. Based on our findings, *S. zeamais* infesting maize grains caused substantial kernel damage, consistent with the studies of Quellhorst et al. [34]. These results suggest that PE storage is an effective method for reducing grain damage, particularly for common beans (NUV6) during storage.

### 3.4. Effect of biosilica on weight loss of common bean and maize seeds

The mean percentage weight losses of maize and common bean seeds treated with biosilica powder over the storage period are shown in Fig 3.

**Fig 3**. Evolution of weight loss for (a) treated maize, (b) untreated maize, (c) treated common bean and (d) untreated common bean seeds.

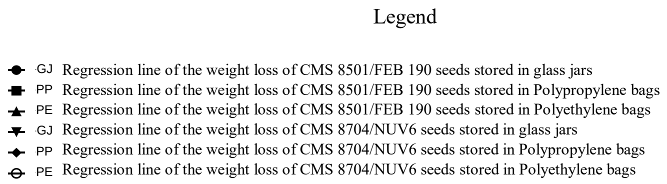


Figs 3a and 3b show weight losses of less than 10% for grains treated and stored in GJs and PP and PE bags over a 6-month period, except for untreated common bean grains in GJs, which exhibited weight losses of 17% and 18% for NUV6 and FEB-190, respectively (Figs 3c and 3d). High weight losses observed in infested, untreated maize and common bean seeds may result from insect infestation consuming the endosperm [35]. Another possible explanation could be the physiological respiration in common beans, which breaks down carbohydrate and protein reserves to produce $CO_2$. The lower weight loss in grains treated and stored in PP and PE bags compared to untreated grains in GJs is likely due to reduced moisture evaporation. Weight loss increased significantly between the first and last months for all treated and untreated grains stored in different containers, likely due to the gradual reduction in biosilica's insecticidal activity as moisture evaporates from the grains, equilibrating with the laboratory environment. Covele et al. [36] also observed a higher percentage loss in PP bags after six months of storage, while Odjo et al. [37] reported the failure of PP bags to minimize weight losses. Maize weight losses in this study are lower than the 20% and 80% reported by Taddese et al. [38] for maize stored traditionally for 3 to 6 months. Our findings show have a better outcome than the work of Muhammad et al. [39] which established 61% and 57% damage on red and black landraces of Bambara groundnut (*Vigna subterranean*) seeds respectively,

following attack by *Callosobruchus maculatus* which translated to weight loss suffered by the affected seeds.

### 3.5. Variation of insect perforation index on treated common bean and maize seeds

The mean perforation indices of stored maize and common bean seeds treated throughout the storage months are displayed in Fig 4.

**Fig 4**. Evolution of perforation index on (a) maize (CMS 8704 and CMS 8501) and (b) common beans (NUV6 and FEB-190) grains.

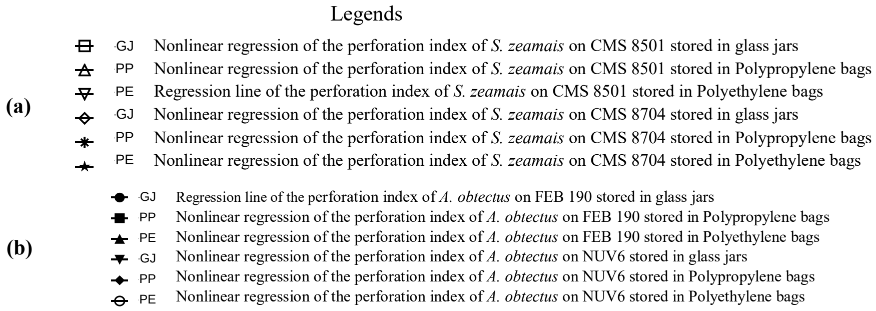


As depicted in Fig 4, insect perforation indices follow a non-linear pattern and tend to plateau between 40 and 50% from the second to the sixth month across all three preservation materials for both corn and common bean kernels. As a rule of thumb, an IPI value lower than 50 indicates a positive protectant effect, while an IPI greater than 50 indicates negative protectability [40]. Fig 4a shows a perforation index ≥ 50% in the first and second month for CMS 8704 kernels stored in PE bags and CMS 8501 kernels stored in PP bags. Similarly, Fig 4b also shows a perforation index ≥ 50 in the 3rd month for FEB-190 common bean grains stored in PE bags. Perforation indices over 50% indicate an increased pest infestation in CMS 8501, CMS 8704 and FEB-190, suggesting low biosilica activity [41]. In contrast, perforation indices ≤ 50% reflect lower grain infestation due to the effectiveness of biosilica against both pests. The overall reduction in perforation indices across the three materials for all grains suggests an ovicidal or even larvicidal effect of biosilica, which prevents insect molting, development, and consequently, emergence.

### 3.6. Viability of maize and commons seeds stored in different materials

The germination rate of seeds treated with biosilica powder and stored in the different items is given in Fig 5.

**Fig 5**. Evolution of viability for (a) maize (CMS 8704 and CMS 8501) and (b) common bean seeds (NUV6 and FEB-190) after 6 months of storage.

Legends

(a)
GJ Nonlinear regression of the viability of maize grains CMS 8501 after 06 months of storage in glass jars
PP Nonlinear regression of the viability of maize grains CMS 8501 after 06 months of storage in Polypropylene bags
PE Nonlinear regression of the viability of maize grains CMS 8501 after 06 months of storage in Polyethylene bags
GJ Nonlinear regression of the viability of maize grains CMS 8704 after 06 months of storage in glass jars
PP Nonlinear regression of the viability of maize grains CMS 8704 after 06 months of storage in Polypropylene bags
PE Nonlinear regression of the viability of maize grains CMS 8704 after 06 months of storage in Polyethylene bags

(b)
GJ Nonlinear regression of the viability of common bean grains FEB 190 after 06 months of storage in glass jars
PP Nonlinear regression of the viability of common bean grains FEB 190 after 06 months of storage in Polypropylene bags
PE Nonlinear regression of the viability of common bean grains FEB 190 after 06 months of storage in Polyethylene bags
GJ Nonlinear regression of the viability of common bean grains NUV6 after 06 months of storage in glass jars
PP Nonlinear regression of the viability of common bean grains NUV6 after 06 months of storage in Polypropylene bags
PE Nonlinear regression of the viability of common bean grains NUV6 after 06 months of storage in Polyethylene bags

Figs 5a and 5b show that germination percentages range from 30% to 80% for maize (CMS 8501 and CMS 8704) grains and from 27% to 91% for common bean (FEB-190 and NUV6) seeds after 7 days of storage in the three different storage materials. The low germination percentages observed in the treated seeds stored in GJs are due to the consumption of the seed endosperm by the insect pests *A. obtectus* and *S. zeamais,* resulting in reduced viability. The endosperm plays a crucial role in germination, providing the nutrients (sugars, proteins, lipids) needed by the seedling to grow until it can produce its own food by photosynthesis. Thus, its consumption by insects renders it non-functional and reduces the seeds' ability to germinate [42]. On the other hand, germination percentages above 50% observed in seeds stored in PP and PE bags can be attributed to the low oxygen permeability of these materials. The limited oxygen inside the bags shortens the lifespan of both insect pests, making it difficult for them to perforate the grains [43].

In this study, common bean seeds treated and stored in PP and PE bags maintained over 85% viability, while maize seeds retained approximately 80% viability after 6 months and 7 days of germination experiments. These results are close to the maximum germination rate of 86% reported by Danga et al. [44] for maize seeds treated with NeemPro®. Biosilica used in this study did not have negative affect maize seed germination. However, no germination was recorded for either common bean seeds or untreated maize grain (negative control) stored for 6 months in GJs, PP and PE bags. This was due to increased infestation by *A. obtectus* and *S. zeamais,* which resulted in the loss of seed viability.

## Conclusion

This study focused on determining grain damage, weight loss, IPI, and grain viability after manually mixing biosilica at doses of 10 g and 2.5 g with 200 g of maize (CMS 8501 and CMS

8704) and common beans (FEB 190 and NUV6), stored in PE and PP bags, as well as 50 g of the same seeds stored in a GJs over a period of 6 months. Grain damage, weight loss, and seed viability were found to be dependent on grain type, storage material and storage duration, with an increase in storage time resulting in significant losses in grain weight and seed viability. PE bags provided a highly efficient, hermetic storage environment compared to PP bags and GJs, leading to better preservation of common bean seeds than maize seeds when mixed with biosilica. Based on these findings, biosilica mixed with grains and stored in PE bags can reduce grain damage and weight loss for up to 3 months without replenishment, and for up to 5 months if biosilica is reapplied after three months. The storability of these seeds can be further extended by placing the PE bag containing biosilica inside ordinary storage bags to create multi-layer PE storage, ensuring a water-resistant and fully airtight storage condition. Adopting these findings as proactive measures during post-harvest grain storage could reduce grain losses and improve livelihoods and incomes of farmers, marketers, and processors in Cameroon. Nevertheless, proximate analyses and surveillance of mycotoxin levels in grains must be conducted after 6 months of storage. Further studies are also necessary to explore encapsulating essential oils on biosilica to extend its effectiveness, and to monitor living insect pest populations to model insect progression and grain damage throughout the storage period. Additionally, tests should be conducted to evaluate the impact of biosilica treatment on the organoleptic and technological qualities of the grains.


## Acknowledgments

Special thanks to the management of the Institute of Agricultural Research for Development (IRAD), Cameroon for their support.


## Author contributions

Proposed methodology, conceptualized, performed statistical analyses, funding acquisition, Formal analysis, investigated the study and wrote the original draft : **BZZ**.
Reviewed and edited the original draft: **CRMM, AAV and LMAM.**
Corrected methodology, contributed to data curation, reviewed and edited the original draft: **EEE**:
Supervised, reviewed and edited the original draft : **PDBB** and **CMK**.


## Funding

We gratefully acknowledge the Royal Society of Chemistry for the Research Fund grant (**R24-9789259332**).

## Consent to participate

Not applicable.

## Consent to publish

All authors read and approved the publishing of this article.

## Competing interest

The authors declare that they have no conflicts of interest.

## Data Availability Statement

The data of this study will be available from the corresponding authors on request.

## Ethics declaration

Not applicable

## References


1. Zing BZ, Messi MLA, Ehabe EE, Belibi DPB, Kede CM. Protection of stored common bean and maize grains using optimally synthesized biosilica from rice husk ash. J. Food Biochem. 2024; 1: 3741615. https://doi.org/10.1155/2024/3741615.

2. OCDE/FAO. Agricultural Outlook 2021-2030, *OECD* Publishing, Paris. 2021: 337. https://doi.org/10.1787/19428846-en.

3. Groote HD, Muteti FN, Bruce AY. On-farm storage loss estimates of maize in Kenya using community survey methods. J Stored Prod Res. 2023; 102: 102107. https://doi.org/10.1016/j.jspr.2023.102107.

4. Darfour B, Rosentrater KA. Cost Assessment of Five Different Maize Grain Handling Techniques to Reduce Postharvest Losses from Insect Contamination. Insects. 2020; 11: 50. https://doi.org/10.3390/insects11010050.

5. Chinji M, Hamabwe S, Kuwabo K, Mugovu I, Thole R, Mazala M, Osorno JM, McClean P, Jochua C, Urrea C, Mukuma C, Chisale V. Kamfwa, K. Introgression and Stability of Common Bean Weevil (*Acanthoscelides obtectus* [Say]) Resistance in Diverse Market Classes From the Andean Gene Pool of Common Bean. Legume Science, 2024; 6: e223. https://doi.org/10.1002/leg3.223

6. Nwosu LC. Chemical bases for maize grain resistance to infestation and damage by the maize weevil, *Sitophilus zeamais* Motschulsky. J Stored Prod Res. 2016; 69:41-50, https://doi.org/10.1016/j.jspr.2016.06.001.

7. Kljajić P, Andrić G, Golić MP, Jovičić I. Bean weevil *Acanthoscelides obtectus (Say)* survival and progeny production affected by residual insecticide deposits, and related damage of two types of beans. J Stored Prod Res. 2022; 98: 102004. https://doi.org/10.1016/j.jspr.2022.102004.

8. Groote HD, Gitonga ZM, Sonder K. Maize storage losses, climate, and climate change in Zambia. Food Secur. 2023; 15: 879-899. https://doi.org/10.1007/s12571-023-01347-7.

9. Baliyarsingh B, Pradhan CK . Prospects of plant-derived metallic nanopesticides against storage pests: A review. J Agric Food Res. 2023; 14: 100687. https://doi.org/10.1016/j.jafr.2023.100687.

10. Bechoff A, Shee A, Mvumi MB, Ngwenyama P, Debelo H, Ferruzzi G, Nyanga M, Mayanja S, Tomlins IK. Estimation of nutritional post-harvest losses along food value chains: A case study of three key food security commodities in Sub-Saharan Africa. Food Secur. 2022; 14: 571-590. https://doi.org/10.1007/s12571-021-01238-9.

11. Cardell L, Michelson H. Price risk and small farmer maize storage in Sub-Saharan Africa: New insights into a long-standing puzzle. Am J Agric Econ. 2023; 105: 735-1045. https://doi.org/10.1111/ajae.12343.

12. Baoua IB, Amadou L, Ousmane B, Baributsa D, Murdock LL. PICS bags for post-harvest storage of maize grain in West Africa. J Stored Prod Res. 2014; 58: 20-28. https://doi.org/10.1016/j.jspr.2014.03.001.

13. Williams SB, Murdock LL, Baributsa D. Storage of maize in purdue improved crop storage (PICS) bags. PLOS ONE. 2017; 12: e0168624. https://doi.org/10.1371/journal.pone.0168624.

14. Walker S, Jaime R, Kagot V, Probst C. Comparative effects of hermetic and traditional storage devices on maize grain: Mycotoxin development, insect infestation and grain quality. J Stored Prod Res. 2018; 77: 34-44. https://doi.org/10.1016/j.jspr.2018.02.002.

15. Ngwenyama P, Mvumi BM, Stathers TE, Nyanga LK, Siziba S. How different hermetic bag brands and maize varieties affect grain damage and loss during smallholder farmer storage. Crop Protec. 2022; 153: 105861. https://doi.org/10.1016/j.cropro.2021.105861.

16. Mohapatra D, Kar A Giri SK. Insect Pest Management in Stored Pulses: an Overview. Food and Bioproc Tech. 2015; 8(2): 239-265.

17. Kavallieratos NG, Michail EJ, Boukouvala MC, Nika EP, Skourti A. Efficacy of pirimiphos-methyl, deltamethrin, spinosad and silicoSec against adults and larvae of Tenebrio molitor L. on wheat, barley and maize. J Stored Prod Res. 2019; 83: 161-167. https://doi.org/10.1016/j.jspr.2019.06.012.

18. Mir SA, Mir MB, Shah MA, Hamdani AM, Sunooj KV, Phimolsiripol Y, Khaneghah AM. New prospective approaches in controlling the insect infestation in stored grains. J. Asia Pac. Entomol. 2023; 26: 102058. https://doi.org/10.1016/j.aspen.2023.102058.

19. Tofel KH, Kosma P, Stähler M, Adler C, Nukenine EN. Insecticidal products from *Azadirachta indica* and *Plectranthus glandulosus* growing in Cameroon for the protection of stored cowpea and maize against their major insect pests. Ind Crops Prod. 2017; 110: 58-64. https://doi.org/10.1016/j.indcrop.2017.09.051.

20. Singh KD, Mobolade AJ, Bharali R, Sahoo D, Rajashekar Y. Main plant volatiles as stored grain pest management approach: A review. J Agric Food Res. 2021; 4: 100127. https://doi.org/10.1016/j.jafr.2021.100127.

21. Moutassem, D., Boubellouta, T., Bellik, Y., Rouis, Z., Kucher, D. E., Utkina, A. O., Kucher, O. D., Mironova, O. A., Kavhiza, N. J., and Rebouh, N. Y. Insecticidal activity of Thymus pallescens de Noë and Cymbogon citratus essential oils against Sitophilus zeamais and Tribolium castaneum. Sci Rep. 2024; 14: 13951. https://doi.org/10.1038/s41598-024-64757-3.

22. Mesele T, Dibaba K, Garbaba CA, Mendesil E. Effectiveness of different storage structures for the management of Mexican bean weevil, *Zabrotes subfasciatus* (Boheman) (Coleoptera: Bruchidae) on stored common bean, *Phaseolus vulgaris* L. (Fabaceae). J Stored Prod Res. 2022; 96: 101928. https://doi.org/10.1016/j.jspr.2022.101928.

23. Gever ER, Echezona BC. The Insecticidal Activity of Neem and Palm Kernel Oils on Bean Weevil (*Callosobruchus maculatus F.)* (Coleoptera: Chrysomelidae: Bruchinae) Infestations of Stored Cowpea (*Vigna unguiculata L. Walp*). J. Food Process. Preserv. 2023; 9: 5536943. https://doi.org/10.1155/2023/5536943.

24. Berhe M, Subramanyam B, Demissie G, Chichaybelu M, Fetien AF, Mahroof R, Harvey J. Effect of storage duration and storage technologies on pest infestations and post-harvest quality loss of stored sesame seeds in Ethiopia. J Stored Prod Res. 2023; 103: 102161. https://doi.org/10.1016/j.jspr.2023.102161.

25. Kläsener GR, Ribeiro ND, Casagrande CR, Arns FD. Consumer preference and the technological and nutritional quality of different bean colours. Acta Sci. Agron. 2018; 42: e43689. https://doi.org/10.4025/actasciagron.v42i1.43689.

26. Ziegler V, Paraginski RT, Ferreira CD. Grain storage systems and effects of moisture, temperature and time on grain quality - A review. J Stored Prod Res. 2021; 91: 101770. https://doi.org/10.1016/j.jspr.2021.101770.

27. Abass BA, Fischler M, Schneider S, Shamim D, Audifas G, Rüst J, Kabula E, Ndunguru G, Madulu D, Msola D. On-farm comparison of different postharvest storage technologies in a maize farming system of Tanzania Central Corridor. J Stored Prod Res. 2018; 77: 55-65. https://doi.org/10.1016/j.jspr.2018.03.002.

28. Győri Z. Chapter 11 - Corn: Grain-Quality Characteristics and Management of Quality Requirements, Editor(s): Wrigley C, Batey I, Miskelly D. In Woodhead Publishing Series in Food Science, Technology and Nutrition, Cereal Grains (Second Edition), Woodhead Publishing. 2017: 257-290. http://dx.doi.org/10.1016/B978-0-08-100719-8.00011-5.

29. Cortese D, de Oliveira GS, Fernandes MG. Resistance evaluation of maize varieties to *Sitophilus zeamais* infestation across two generations: Insights for Integrated Pest Management. J Stored Prod Res. 2024; 109: 102473. https://doi.org/10.1016/j.jspr.2024.102473.

30. Suleiman R, Rosentrater KA, Bern CJ. Evaluation of maize weevils *Sitophilus zeamais Motschulsky* infestation on seven varieties of maize. J Stored Prod Res. 2015; 64: 97-102, https://doi.org/10.1016/j.jspr.2015.09.005.

31. Vendi T, Aulicky R, Stejkal V. Comparison of the mandible morphology of two stored product bostrichid beetles *Rhyzopertha dominica* and *Prostephanus truncatus*. In: Adler CS, Opit G et al., (Eds), Proceedings of the 12th International Working Conference on Stored Product Protection Berlin Germany 7-11 October 2018, Berlin, Germany, Julius Kuhn Institute. 2018; 208-211. https://doi.org/10.5073/jka.2018.463.049.

32. Misra S, Murmu SB, Debnath S. Coating treatments on jute fabrics for improving their functionality and minimizing the storage losses of grains: A review. Ind Crops Prod. 2024; 216: 118765. https://doi.org/10.1016/j.indcrop.2024.118765.

33. Kuyu CG, Yetenayet BT, Ali M, Aresawum M, Mpagalile JJ. Evaluation of different grain storage technologies against storage insect pests over an extended storage time. J Stored Prod Res. 2022; 96: 101945. https://doi.org/10.1016/j.jspr.2022.101945.

34. Quellhorst HE, Arthur FH, Bruce A, Zhu KY, Morrison WR III. Exposure to a Novel Insecticide Formulation on Maize and Concrete Reduces Movement by the Stored Product Pests, *Prostephanus truncatus (Horn)* and *Sitophilus zeamais (Motschulsky)*. Front agron. 2022; 4: 868509. https://doi.org/10.3389/fagro.2022.868509.

35. Ogbonnaya E, Anigo KM, Bala SM, Muhammad A, Bamaiyi LJ, Precious E, Oluchukwu IU. Assessment of the nutritional content of cowpea seed exposed to plant-derived (azadirachtin, myristicin and α-humulene) insecticides against *Callosobruchus maculatus*. J Agric Food Res. 2024; 16: 101130. https://doi.org/10.1016/j.jafr.2024.101130.

36. Covele G, Gulube A, Tivana L, Ribeiro-Barros AI, Carvalho MO, Ndayiragije A, Nguenha R. Effectiveness of hermetic containers in controlling paddy rice (*Oryza sativa* L.) storage insect pests. J Stored Prod Res. 2020; 89: 101710. https://doi.org/10.1016/j.jspr.2020.101710.

37. Odjo S, Palacios-Rojas N, Burgueño J, Corrado M, Verhulst N. Hermetic storage technologies preserve maize seed quality and minimize grain quality loss in smallholder farming systems in Mexico. J Stored Prod Res. 2022; 96: 101954, https://doi.org/10.1016/j.jspr.2022.101954.

38. Taddese M, Dibaba K, Bayissa W, Hunde D, Mendesil E, Kassie M, Mutungi C, Tefera T. Assessment of quantitative and qualitative losses of stored grains due to insect infestation in Ethiopia. J Stored Prod Res. 2020; 89: 101689. https://doi.org/10.1016/j.jspr.2020.101689.

39. Muhammad I, Abdullahi N, Shu'aibu AB, Musa I, Gambo BZ, Adamu ZA. Effect of cowpea seed beetle (*Callosobruchus maculatus*) infestation on selected landraces of Bambara groundnut during storage. Biorem Sci Technol Res. 2021; 9: 31-34. https://doi.org/10.54987/bstr.v9i2.624.

40. Ashamo MO, Ileke KD, Ogungbite, OC. Entomotoxicity of some agro-wastes against cowpea bruchid, *Callosobruchus maculatus* (Fab.) [Coleoptera: Chrysomelidae]

infesting cowpea seeds in storage. Heliyon. 2021; 7: e07202, https://doi.org/10.1016/j.heliyon.2021.e07202.

41. Ileke KD, Adesina JM, Nwosu LC, Olagunju A. Perforation index assessment of cowpea seeds against cowpea bruchid, *Callosobruchus maculatus* (Fabricius) [Coleoptera: Chrysomelidae], infestation using *Piper guineense*. J. Basic Appl. Zool. 2020; 81: 60. https://doi.org/10.1186/s41936-020-00195-7.

42. Otitodun GO, Ogundare MO, Ajao SK, Nwaubani SI, Abel GI, Opit GP, Bingham G, Omobowale MO. Efficacy of phosphine and insect penetration ability in ZeroFly® bags. J Stored Prod Res. 2019; 82: 81-90. https://doi.org/10.1016/j.jspr.2019.04.006.

43. Yewle N, Swain KC, Mann S, Guru PN. Performance of hermetic bags in green gram [Vignaradiata (L.) R. Wilczek] storage for managing pulse beetle (*Callosobruchus chinensis*). J Stored Prod Res. 2022; 95: 101896, https://doi.org/10.1016/j.jspr.2021.101896.

44. Danga SPY, Nukenine EN, Fotso GT, Adler C. Use of NeemPro®, a neem product to control maize weevil *Sitophilus zeamais* (Motsch.) (Coleoptera: Curculionidae) on three maize varieties in Cameroon. Agric. Food Secur. 2015; 4: 1-7.

Figures Click here to access/download;Figure;Figures.docx

# Efficiency of Biosilica on Grain Losses for Eco-conservation of Maize (*Zea mays* L.) and Common Bean (*Phaseolus vulgaris* L.) Seeds

Bertrand Zing Zing [1,2*], Charles Rostand Mvongo Mvodo [1], Valteri Audrey Voula [1], Lin Marcellin Messi Ambassa [1], Eugene Ejolle Ehabe [1], Placide Desiré Belibi Belibi [3], Charles Melea Kede [2]

[1] Directorate of Scientific Research, Institute of Agricultural Research for Development, P.O. Box 2123, Yaoundé, Cameroon.

[2] Laboratory of Chemical and Industrial Bioprocess Engineering, National Higher Polytechnic School of Douala, University of Douala, P.O. Box 2701, Douala, Cameroon.

[3] Department of Inorganic Chemistry, University of Yaoundé I, P.O. Box 812, Yaoundé, Cameroon.

∗ Corresponding author e-mail address: zingbertrand29@gmail.com (B.Z.Z)

Bertrand Zing Zing: https://orcid.org/0000-0002-3892-8950.

Eugene Ejolle Ehabe: https://orcid.org/0000-0003-2215-2112.

Charles Melea Kede: https://orcid.org/0000-0002-4951-3152.

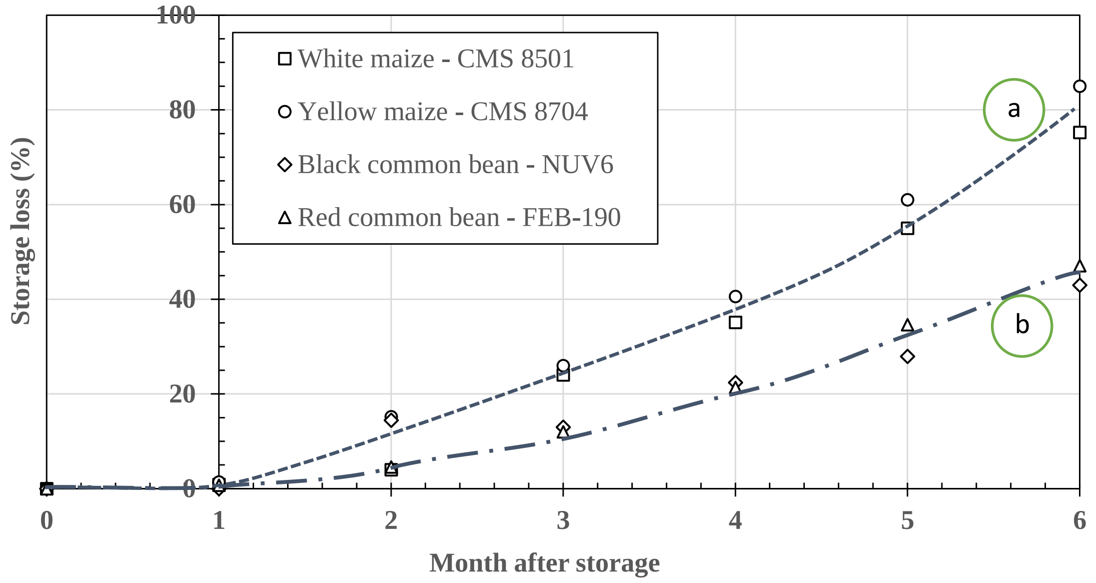


**Fig 1**. Evolution of storage losses for (a) maize and (b) common beans cultivars during 6 months on glass jars in the laboratory.

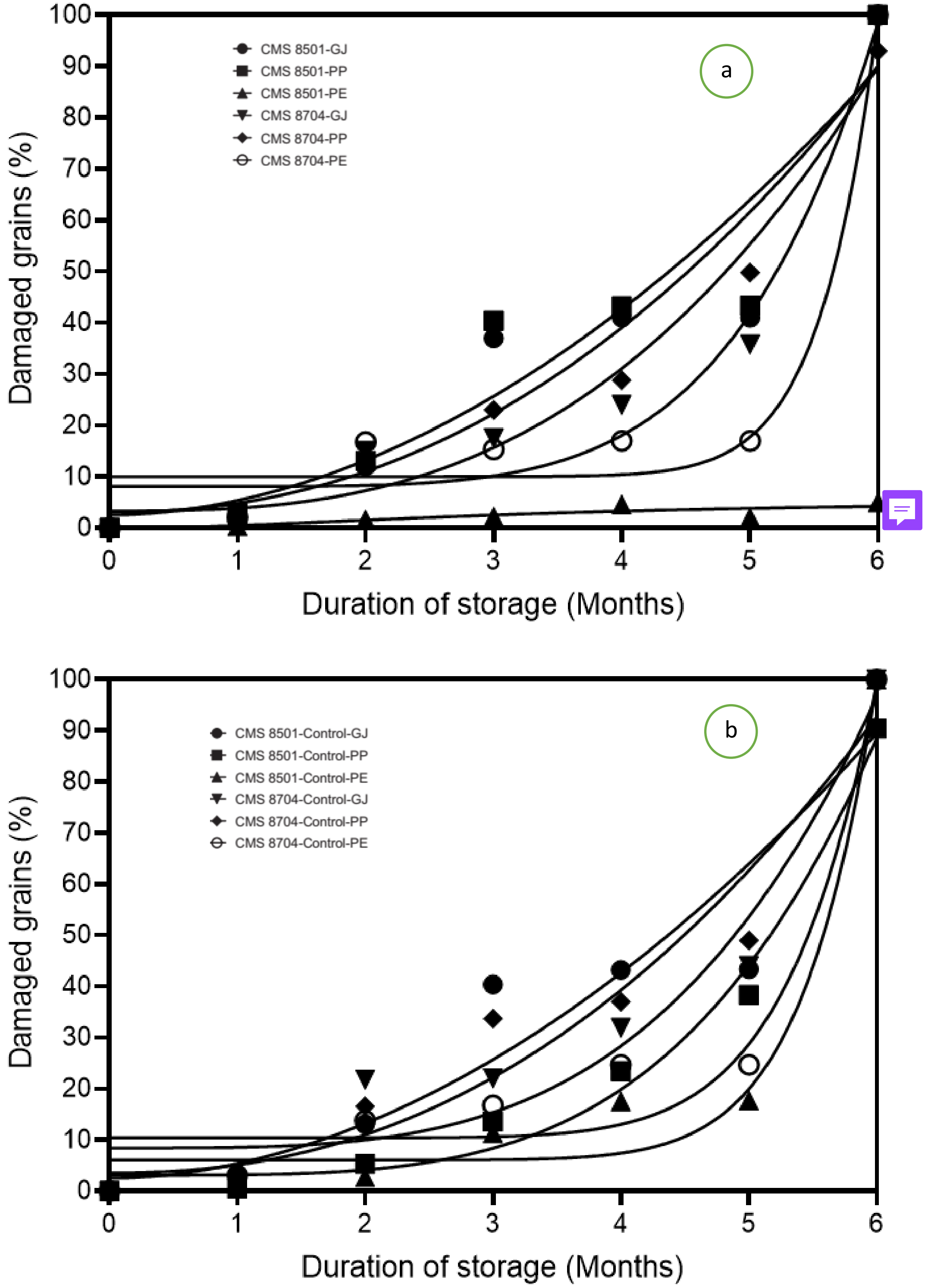

a
CMS 8501-GJ
CMS 8501-PP
CMS 8501-PE
CMS 8704-GJ
CMS 8704-PP
CMS 8704-PE
Damaged grains (%)
Duration of storage (Months)
b
CMS 8501-Control-GJ
CMS 8501-Control-PP
CMS 8501-Control-PE
CMS 8704-Control-GJ
CMS 8704-Control-PP
CMS 8704-Control-PE
Damaged grains (%)
Duration of storage (Months)

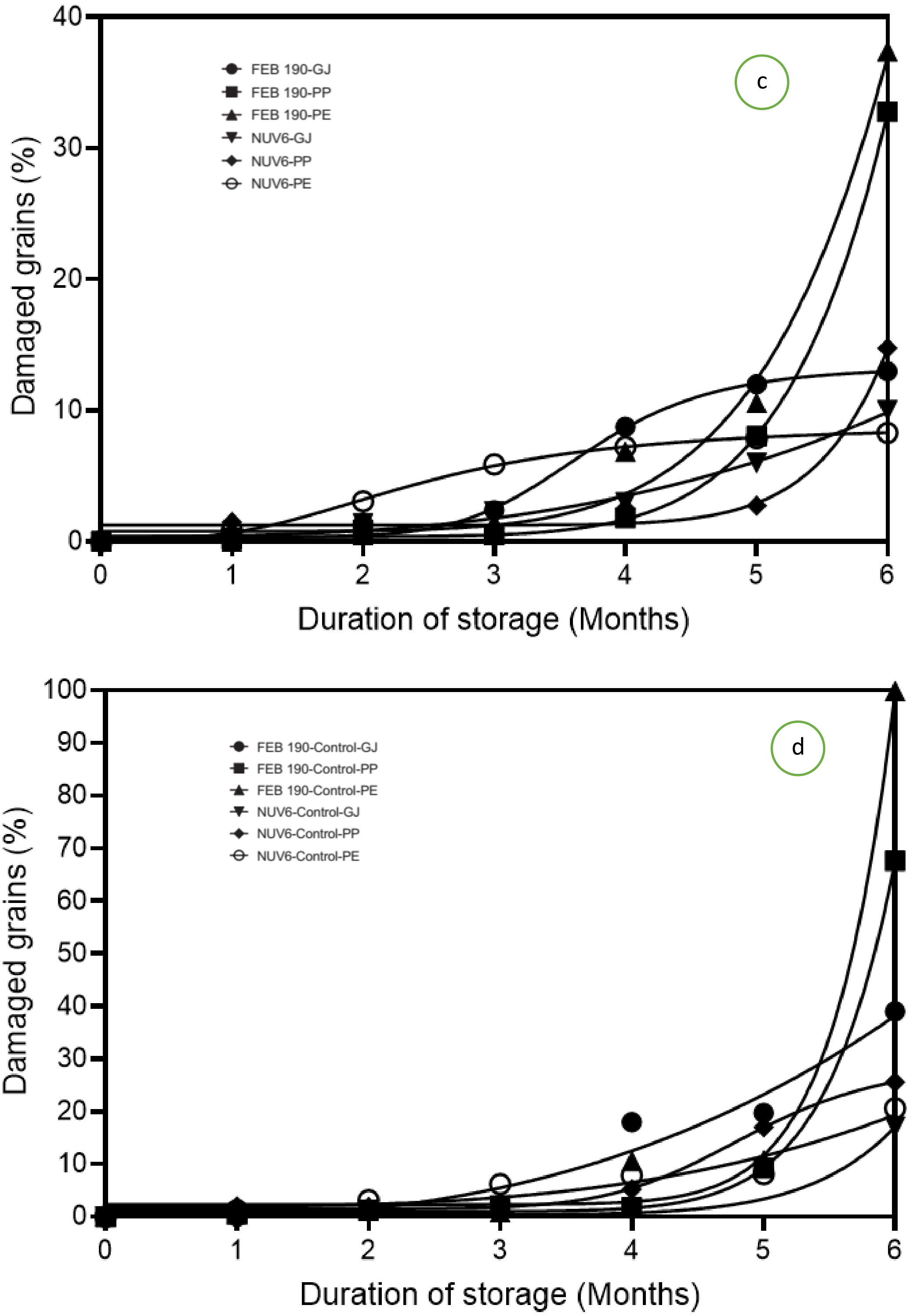


**Fig 2.** Evolution of damaged grains for (a) treated maize, (b) untreated maize, (c) treated common bean and (d) untreated common bean seeds.

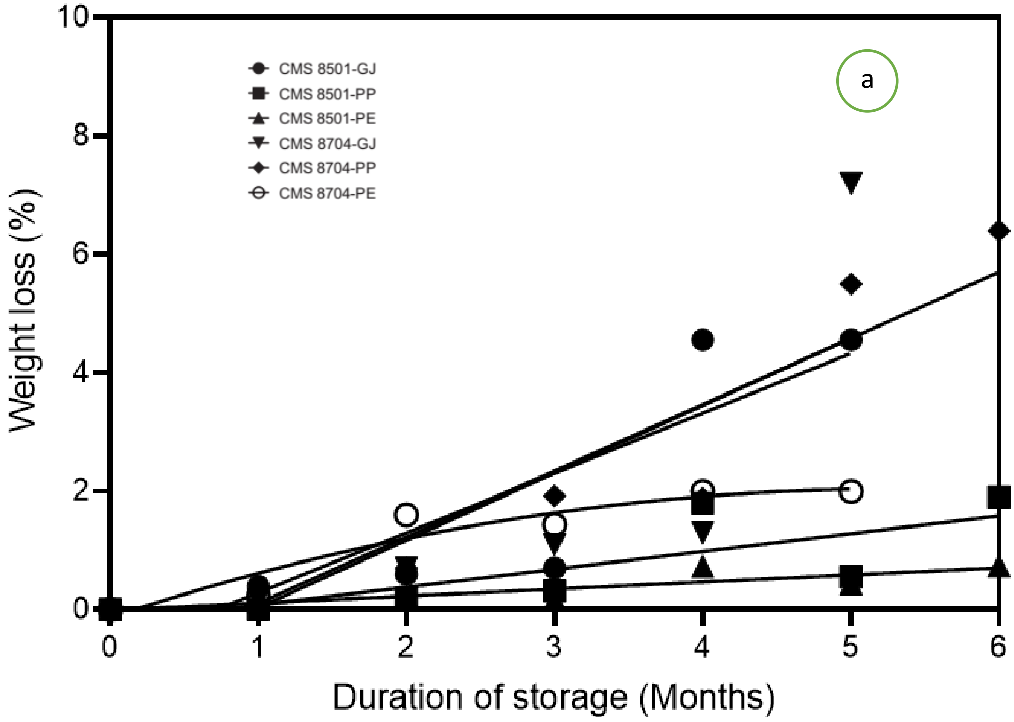

a
CMS 8501-GJ
CMS 8501-PP
CMS 8501-PE
CMS 8704-GJ
CMS 8704-PP
CMS 8704-PE
Weight loss (%)
Duration of storage (Months)
0
2
4
6
8
10
0
1
2
3
4
5
6


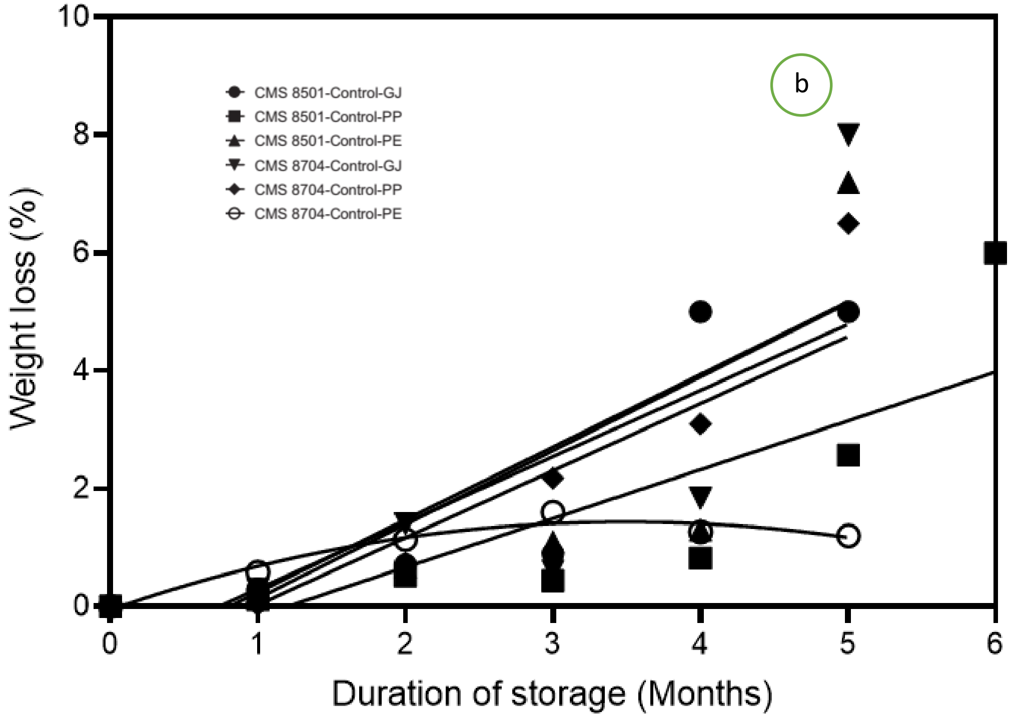

b
CMS 8501-Control-GJ
CMS 8501-Control-PP
CMS 8501-Control-PE
CMS 8704-Control-GJ
CMS 8704-Control-PP
CMS 8704-Control-PE
Weight loss (%)
Duration of storage (Months)
0
2
4
6
8
10
0
1
2
3
4
5
6

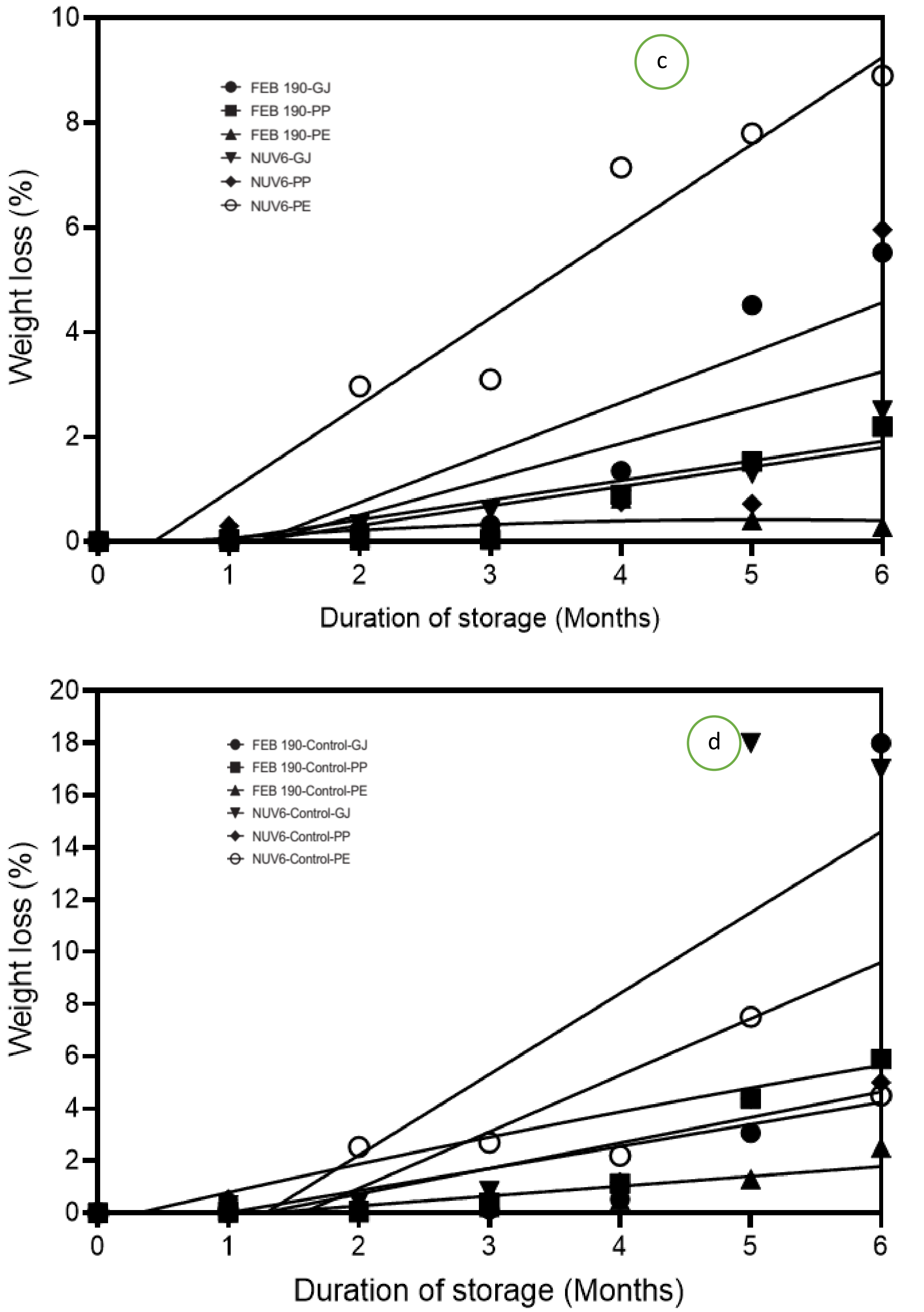


**Fig 3**. Evolution of weight loss for (a) treated maize, (b) untreated maize, (c) treated common bean and (d) untreated common bean seeds.

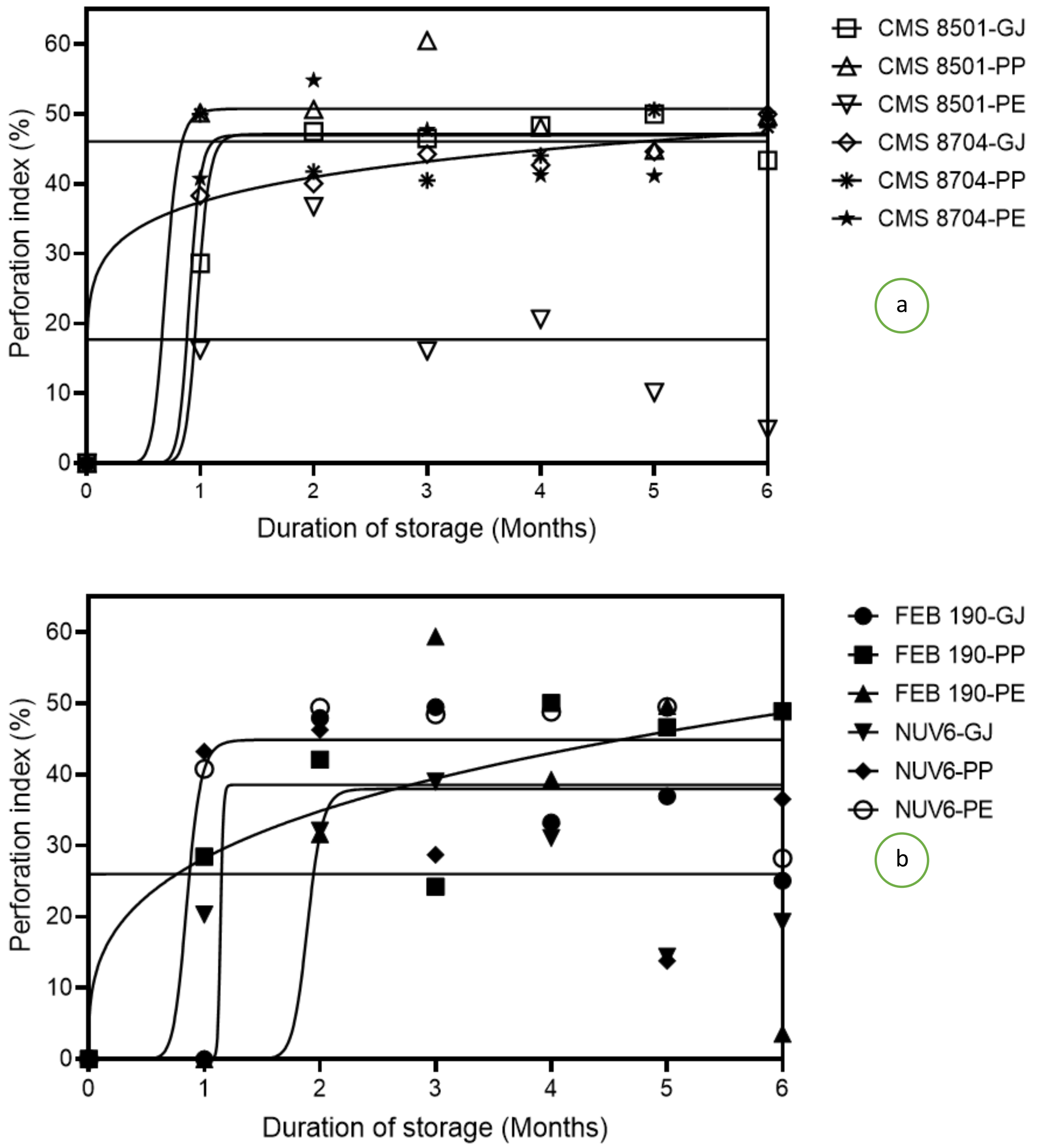


**Fig 4**. Evolution of perforation index on (a) maize (CMS 8704 and CMS 8501) and (b) common beans (NUV6 and FEB-190) grains.

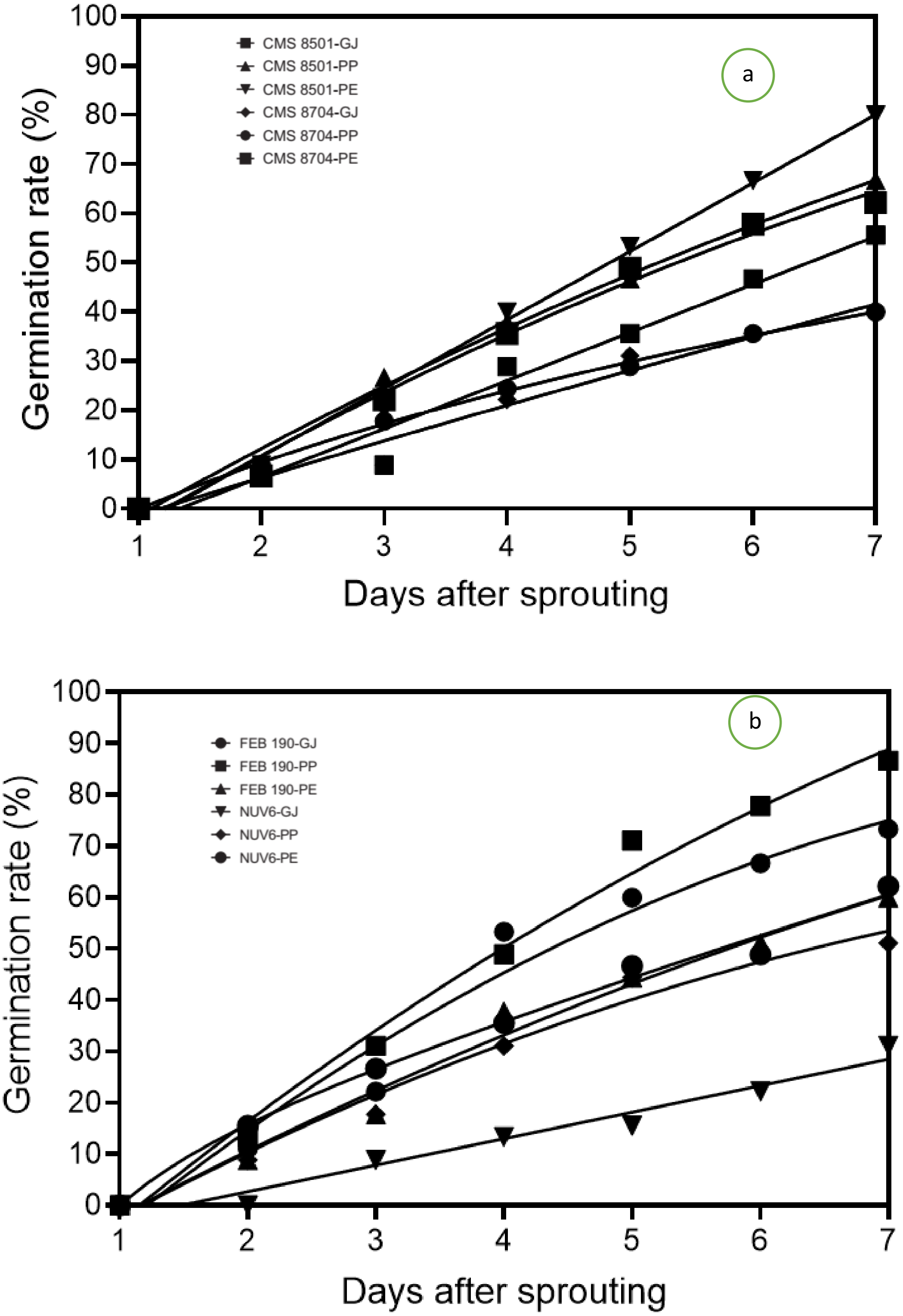


**Fig 5**. Evolution of viability for (a) maize (CMS 8704 and CMS 8501) and (b) common bean seeds (NUV6 and FEB-190) after 6 months of storage.

CMS 8501-GJ
CMS 8501-PP
CMS 8501-PE
CMS 8704-GJ
CMS 8704-PP
CMS 8704-PE

CMS 8501-Control-GJ
CMS 8501-Control-PP
CMS 8501-Control-PE
CMS 8704-Control-GJ
CMS 8704-Control-PP
CMS 8704-Control-PE

Click here to access/download
**Supporting Information**
Supporting information.docx
